# TRIM22 induces cellular senescence by targeting PHLPP2 in hepatocellular carcinoma

**DOI:** 10.1038/s41419-024-06427-w

**Published:** 2024-01-10

**Authors:** Donghee Kang, Hyun Jung Hwang, Yurim Baek, Jee Young Sung, KyeongJin Kim, Heon Joo Park, Young-Gyu Ko, Yong-Nyun Kim, Jae-Seon Lee

**Affiliations:** 1 Research Center for Controlling Intercellular Communication, College of Medicine, Inha University, Incheon, 22212 Korea; 2 Program in Biomedical Science & Engineering, Inha University, Incheon, 22212 Korea; 3 Department of Molecular Medicine, College of Medicine, Inha University, Incheon, 22212 Korea; 4 Metastasis Branch, Division of Cancer Biology, National Cancer Center, Goyang, 10408 Korea; 5 Department of Microbiology, College of Medicine, Inha University, Incheon, 22212 Korea; 6 Division of Life Sciences, Korea University, Seoul, 02841 Korea

**Keywords:** Tumour-suppressor proteins, Senescence

## Abstract

The ubiquitin-proteasome system is a vital protein degradation system that is involved in various cellular processes, such as cell cycle progression, apoptosis, and differentiation. Dysregulation of this system has been implicated in numerous diseases, including cancer, vascular disease, and neurodegenerative disorders. Induction of cellular senescence in hepatocellular carcinoma (HCC) is a potential anticancer strategy, but the precise role of the ubiquitin-proteasome system in cellular senescence remains unclear. In this study, we show that the E3 ubiquitin ligase, TRIM22, plays a critical role in the cellular senescence of HCC cells. TRIM22 expression is transcriptionally upregulated by p53 in HCC cells experiencing ionizing radiation (IR)-induced senescence. Overexpression of TRIM22 triggers cellular senescence by targeting the AKT phosphatase, PHLPP2. Mechanistically, the SPRY domain of TRIM22 directly associates with the C-terminal domain of PHLPP2, which contains phosphorylation sites that are subject to IKKβ-mediated phosphorylation. The TRIM22-mediated PHLPP2 degradation leads to activation of AKT-p53-p21 signaling, ultimately resulting in cellular senescence. In both human HCC databases and patient specimens, the levels of TRIM22 and PHLPP2 show inverse correlations at the mRNA and protein levels. Collectively, our findings reveal that TRIM22 regulates cancer cell senescence by modulating the proteasomal degradation of PHLPP2 in HCC cells, suggesting that TRIM22 could potentially serve as a therapeutic target for treating cancer.

## Introduction

Hepatocellular carcinoma (HCC) is the primary subtype of liver cancer, accounting for ~90% of all cases. HCC is associated with high rates of tumor recurrence and metastasis after primary hepatic resection, contributing to the most common cause of cancer-related mortality worldwide [1, 2]. Radiotherapy (RT) is a major modality used in treating HCC, particularly progressive HCC patients with tumors that are not amenable to resection or transplantation, or those with extrahepatic metastasis tumors [2–4]. However, the efficacy of RT is limited by the endogenous and therapy-induced radioresistance of HCC [5–9]. To improve the effectiveness of RT in the treatment of HCC, there is still a need to identify potential therapeutic targets associated with HCC radioresistance.

Cellular senescence is a state of stable cell cycle arrest characterized by changes in morphology, macromolecule compositions, and the acquisition of pro-inflammatory phenotypes. Senescence can be induced by a variety of endogenous and exogenous stressors, such as telomere shortening, mitochondrial dysfunction, DNA damage, and oncogene activation. Moreover, cancer therapies, including chemotherapy, radiotherapy, and targeted therapy, can trigger therapy-induced senescence (TIS). The induction of cellular senescence is considered as a potential strategy for treating cancer by inducing tumor suppression and immune surveillance [10–12]. However, senescent cancer cells acquire pro-tumorigenic properties through activation of the senescence-associated secretory phenotype (SASP), which can modulate the tumor microenvironment and increase cancer stemness, invasion, migration, angiogenesis, and immune evasion [7, 8]. Nonetheless, the induction of cellular senescence remains a promising strategy for combination therapies in cancer treatment. Recent studies have revealed that pro-senescence therapy can increase the vulnerability of tumors to combination treatments, particularly with the use of senolytics and senomorphic agents. This approach provides new avenues for enhancing treatment outcomes and addressing challenges related to senescence-associated phenotypes [13–15].

The ubiquitin-proteasome system (UPS) is an important protein homeostasis mechanism that targets substrates for ubiquitin-mediated degradation. This process is mediated by three enzymes: a ubiquitin activating enzyme (E1), a ubiquitin conjugating enzyme (E2), and a ubiquitin ligase (E3) [16]. Dysregulation of E3 ligases is commonly observed in cancer: Changes in E3 ligase expression or activity can affect the progression, development, immune checkpoint regulation, and drug responses of various cancers [17, 18]. E3 ligases play a crucial role in determining the specificity and selectivity of ubiquitinated substrates, and they can have oncogenic or tumor-suppressive properties depending on their substrates [19–24]. Understanding the roles of E3 ligases in cancers can provide valuable insights into treating the disease. The activity of oncogenic E3 ligases can be inhibited by small molecules or peptides, such as PROTAC (proteolysis targeting chimeric) or molecular glues. For tumor-suppressive E3 ligases, on the other hand, potential therapeutic strategies include reinstating their expression or activity, exploring synthetic lethality, or targeting downstream oncogenic substrates [17, 18]. E3 ligases contribute to cellular senescence by suppressing or promoting DNA damage responses and cell cycle arrest [25–28]. The E3 ligases tripartite motif-containing 22 (TRIM22) is a TRIM protein family member and is characterized by the presence of RING, BBox, and Coiled-Coil domain regions at the N-terminus and a SPRY region at the C-terminus. TRIM22 has been implicated in regulating the progression and development of various cancers, including glioblastoma, osteosarcoma, and gastric cancer [29–31]. However, the potential role of TRIM22 in the cellular senescence of HCC remains completely unexplored.

In this study, we demonstrate that TRIM22 is upregulated by p53 and its overexpression attenuates the AKT phosphatase PHLPP2 via the UPS, resulting in activation of AKT-p53-p21 senescence pathway. Our study suggests that TRIM22 can be a promising target for the cancer treatment.

## Results

### TRIM22 induces cellular senescence in HCC

**Fig. 1:**
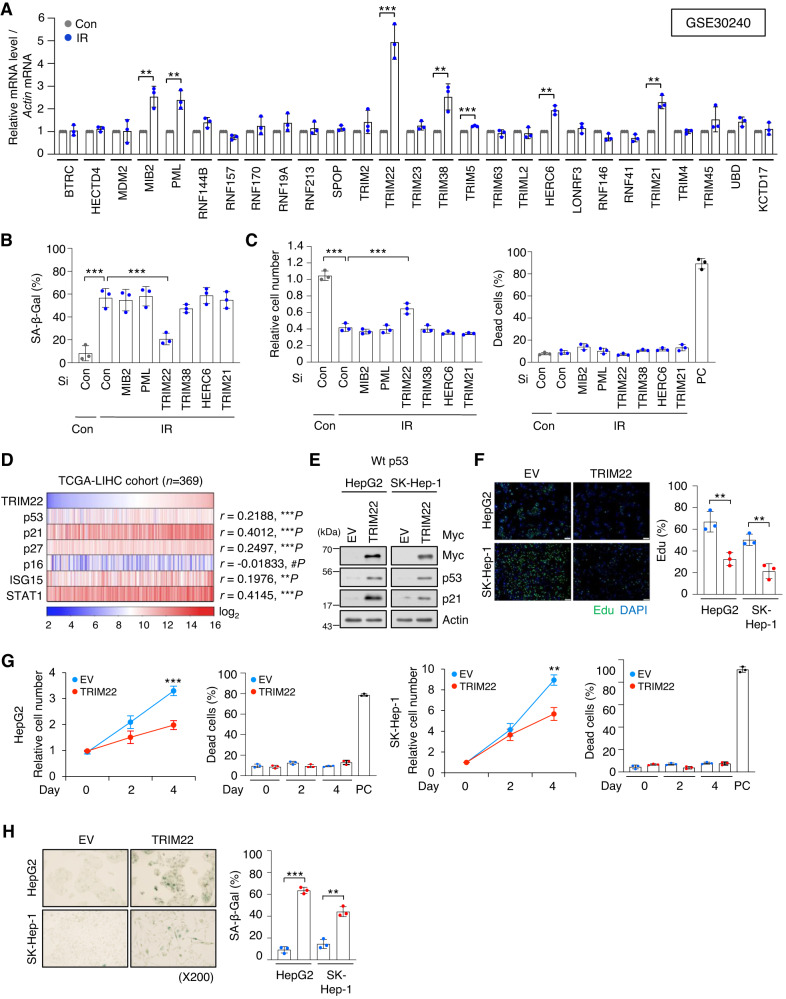
Upregulation of TRIM22 induces cellular senescence in HCC. **A** RT-qPCR of an expression profiling array (GSE30240) for IR-treated HepG2 cells. Data are presented as mean ± SD (unpaired two-tailed *t*-test, MIB2: **P = 0.0044, *t* = 5.805; PML: **P = 0.0042, *t* = 5.874; TRIM22: ***P = 0.0008, *t* = 8.990; TRIM38: **P = 0.01, *t* = 4.606; TRIM5: ***P = 0.0001, *t* = 14.34; HERC6: **P = 0.0013, *t* = 8.116; TRIM21: **P = 0.0016, *t* = 7.667; *n* = 3). **B, C** HepG2 cells were transfected with specific siRNA against each E3 ligase candidate in HepG2 cells. SA-β-Gal assay (**B**) (one-way ANOVA with Tukey’s multiple comparison test, F(7,16) = 21.96, ***P < 0.0001; ***P = 0.0003, *n* = 3) and cell counting (**C**) were performed. Data are presented as mean ± SD (one-way ANOVA with Tukey’s multiple comparison test, F(7,16) = 92.09, ***P < 0.0001; ***P = 0.0002, *n* = 3). Positive control (PC) for dead cells, Doxorubicin 2 μg/mL. **D** Gene expression analysis of TRIM22 and senescence-associated genes in the TCGA-LIHC database (Pearson correlation, *n* = 369). **E**–**H** Western blotting (**E**), Edu incorporation assay. Scale bars, 100 μm (**F**) Data are presented as mean ± SD (unpaired two-tailed *t*-test, HepG2: **P = 0.0059, *t* = 5.352, *n* = 3; SK-Hep-1: **P = 0.0046, *t* = 5.724, *n* = 3), cell counting (**G**) Data are presented as mean ± SD (unpaired two-tailed *t*-test, HepG2: ***P = 0.0008, *t* = 9.097, *n* = 3; SK-Hep-1: **P = 0.0020, *t* = 7.168, *n* = 3), and SA-β-Gal assay (**H**) Data are presented as mean ± SD (unpaired two-tailed *t*-test, HepG2: ***P < 0.0001, *t* = 23.00, *n* = 3; SK-Hep-1: **P = 0.0012, *t* = 8.265, *n* = 3) were performed in TRIM22-overexpressing HepG2 and SK-Hep-1 HCC cells.

To identify E3 ligases that contribute to the therapy-induced senescence of HCC cells, we analyzed an expression profiling array (GSE30240) for IR (ionizing radiation)-induced senescent HepG2 cells. We found that 26 E3 ligases were upregulated (fold change > 2) after IR treatment. When we validated these findings by RT-qPCR, we found that the mRNAs encoding MIB2, PML, TRIM22, TRIM38, HERC6, and TRIM21 were increased by more than 1.5 times in IR-treated HepG2 cells (Fig. 1A). To investigate which E3 ligases play critical roles in the cellular senescence of HCC, we transfected small interfering RNA (siRNA, Si) to knockdown each of the identified E3 ligases in IR-treated HepG2 cells, and monitored typical senescent traits. Only TRIM22 depletion was found to reduce SA-β-Gal positivity (Fig. 1B) and partially rescue the cell number (Fig. 1C) in IR-treated HepG2 cell cultures. We analyzed TRIM22 expression in HCC cell lines with wild-type p53 (Wt p53) (SK-Hep-1, HepG2) or mutant p53 (Mut p53) (SNU449, Huh7, PLC/PRF/5). TRIM22 was found to be markedly upregulated in HCC cells with Wt p53, but not Mut p53, after IR treatment (Fig. S1A). To verify whether IR induces cell death in HCC cell lines with Wt p53 (SK-Hep-1 and HepG2), we conducted Western blot and caspase-3 activity analyses. The results revealed that the protein levels of p53 and p21 were increased in both SK-Hep-1 and HepG2 after IR treatment (Fig. S1B). However, neither cleaved PARP (C-PARP) nor caspase-3 activity was increased in IR-treated SK-Hep-1 and HepG2 cells, indicating that IR did not induce cell death in SK-Hep-1 and HepG2 cells (Fig. S1B–D). Next, we analyzed TCGA data and found that TRIM22 expression was higher in Wt p53 HCC tissues compared to Mut p53 HCC tissues (Fig. S1E). Furthermore, knockdown of p53 in HepG2 cells resulted in the downregulation of TRIM22 at mRNA and protein levels (Fig. S1F). A chromatin immunoprecipitation (ChIP) assay performed with a p53 antibody in IR-treated HepG2 cells revealed that p53 directly bound to the p53-response element in the intron 1 of TRIM22, and its binding affinity was enhanced upon IR treatment (Fig. S1G). These findings indicated that TRIM22 is positively regulated by Wt p53, which directly binds to the p53-response element in the intron 1 of TRIM22.

Next, we analyzed the correlation between TRIM22 and senescence-associated genes in the TCGA-LIHC database and found that p53, p21, p27, ISG15, and STAT1 were positively correlated with upregulated TRIM22 expression in HCC patient samples (Fig. 1D). These data suggested that TRIM22 might function as an upstream regulator of cellular senescence. To explore the possible biological function of TRIM22 in cellular senescence, we overexpressed TRIM22 in two HCC cell lines: HepG2 and SK-Hep-1 cells. Western blotting analysis confirmed that p53 and p21 are increased in TRIM22-overexpressed HCC cells (Fig. 1E). We observed that TRIM22 overexpression reduced cell proliferation, as indicated by Edu incorporation assay and cell counting (Fig. 1F, G). However, PARP cleavage and caspase-3 activity did not increase in both HepG2 and SK-Hep-1 cells following TRIM22 overexpression (Fig. S2A–D). TRIM22 overexpression decreased cell proliferation without causing cell death (Fig. 1F, G, Fig. S2A–D). Additionally, senescence-associated β-galactosidase (SA-β-Gal) positivity was increased in TRIM22-overexpressed HCC cells (Fig. 1H). Taken together, these results suggest that TRIM22 induces cellular senescence by activating the p53-p21 signaling pathway in HCC.

### TRIM22 modulates AKT phosphorylation through degradation of PHLPP2

**Fig. 2:**
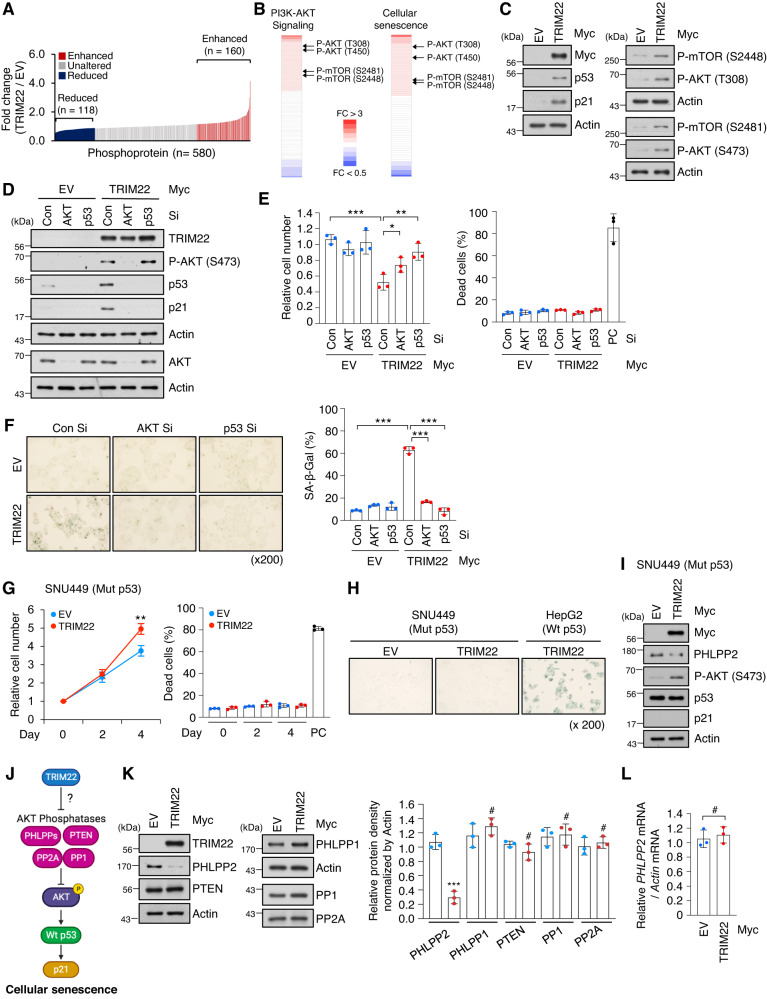
TRIM22 activates AKT-p53-p21 signaling by reducing the PHLPP2 protein level. **A** Phosphoprotein array analysis showing the fold change of phosphoproteins upon TRIM22 overexpression in HepG2 cells. The level of each phosphoprotein was normalized to the total protein level; those above 1.2-fold (160 proteins) and below 0.8-fold (118 proteins) were labeled in red and blue, respectively. **B** Heatmaps representing the phosphorylation sites of proteins enriched in PI3K/AKT signaling or cellular senescence. **C** Western blot analysis of HepG2 cells transfected with empty vector (EV) or TRIM22-expressing vector, as generated using the indicated antibodies. **D**–**F** HepG2 cells were transfected with Con Si, AKT Si, or p53 Si, and then transfected with EV or TRIM22-expressing vector. Western blotting (**D**), relative cell number (**E**) Data are presented as mean ± SD (one-way ANOVA with Tukey’s multiple comparison test, F(5,12) = 16.37, ***P < 0.0001; *P = 0.0314; **P = 0.0015, *n* = 3), and SA-β-Gal positivity (**F**) Data are presented as mean ± SD (one-way ANOVA with Tukey’s multiple comparison test, F(5,12) = 130.2, ***P < 0.0001; ***P < 0.0001; ***P < 0.0001, *n* = 3) were analyzed. Positive control (PC) for dead cells, Doxorubicin 2 μg/mL. **G**–**I** SNU449 HCC cells (Mut p53) were transfected with EV or TRIM22-expressing vector. Cell counting (**G**) Data are presented as mean ± SD (unpaired two-tailed *t-*test, **P = 0.0072, *t* = 5.066, *n* = 3), SA-β-Gal s*t*aining (**H**), and Western blotting (**I**) were performed. PC for dead cells, Doxorubicin 2 μg/mL. **J** Schematic representation of possible means by which TRIM22 induces cellular senescence through the AKT-p53-p21 pathway. **K** Western blot analysis of TRIM22-overexpressed HepG2 cells using the indicated antibodies (left). Phosphatase levels were quantified from protein bands and are presented as mean values of the ratio relative to the levels in EV-transfected HepG2 cells (right). Actin was used as an endogenous control for protein level normalization. Data are presented as mean ± SD (unpaired two-tailed *t*-test, PHLPP2: ***P = 0.0006, *t* = 9.806, *n* = 3; PHLPP1: ^#^*P* = 0.3344, *t* = 1.097, *n* = 3; PTEN: ^#^*P* = 0.1780, *t* = 1.632, *n* = 3; PP1: ^#^*P* = 0.8012, *t* = 0.2690, *n* = 3; PP2A: ^#^*P* = 0.5672, *t* = 0.6227, *n* = 3). **L** RT-qPCR of PHLPP2 mRNA in TRIM22-overexpressed HepG2 cells. Data are presented as mean ± SD (unpaired two-tailed *t-*test, ^#^*P* = 0.6045, *t* = 0.5613, *n* = 3).

To explore the upstream signaling pathway of p53-p21 in TRIM22-mediated HCC senescence, we applied phosphoprotein array analysis. Our results showed that the phosphorylation levels of 278 proteins were changed by TRIM22 overexpression; proteins showing levels with changes of more than 1.2-fold (160 proteins) and less than 0.8-fold (118 proteins) were included for further analysis (Fig. 2A). We found that these phosphorylated proteins were involved in the PI3K-AKT signaling and cellular senescence pathways (Fig. 2B). Increased phosphorylation levels of AKT (T308 and S473) and mTOR (S2448 and S2481) were confirmed by Western blot analysis of TRIM22-overexpressed HepG2 cells (Fig. 2C).

We next investigated whether AKT and p53 are critical as downstream molecules for TRIM22-mediated cellular senescence. Our results revealed that depletion of AKT or p53 decreased p21 accumulation and SA-β-Gal positivity and rescued cell proliferation in TRIM22-overexpressed HepG2 cells (Fig. 2D–F). However, overexpression of TRIM22 in Mut p53 SNU449 cells failed to induce cellular senescence and did not cause cell death (Fig. 2G–I, Fig. S2E, F). These results indicate that AKT and p53 are critical downstream players in TRIM22-mediated cellular senescence.

We hypothesized that the ability of the E3 ligase, TRIM22, to increase AKT phosphorylation could reflect a decrease in the levels and/or activities of AKT phosphatases, such as PHLPPs (PHLPP1 and PHLPP2), PTEN, PP1, and PP2A (Fig. 2J). We examined level changes of these phosphatases and found that TRIM22-overexpressed cells exhibited a specific decrease of PHLPP2 at the protein level, with no change in the mRNA level (Fig. 2K, L). These results suggest that TRIM22 increases AKT phosphorylation by reducing the protein level of the AKT phosphatase, PHLPP2.

Two members of the PHLPP family, PHLPP1 and PHLPP2, function to dephosphorylate AKT [32]. To further investigate the involvement of PHLPP1 and/or PHLPP2 in AKT activation for HCC senescence, we depleted PHLPP1 and PHLPP2 by using each specific siRNA (Si). These knockdown studies revealed that PHLPP2 specifically activated AKT-p53-p21 signaling, whereas PHLPP1 did not affect this signaling (Fig. S3A). Cells with depletion of PHLPP2 showed decreased cell proliferation and increased SA-β-Gal positivity, whereas such senescence traits were not in observed in PHLPP1-depleted cells (Fig. S3B, C). These results indicate that PHLPP2, but not PHLPP1, contributes to TRIM22-mediated cellular senescence as a downstream regulator of TRIM22 and an upstream regulator of AKT. A previous study [33] reported that PHLPP2 expression is suppressed by Mut p53 in colorectal cancer. To investigate the expression of PHLPP2 in HCC cell lines, we analyzed the Cancer Cell Line Encyclopedia (CCLE)-Liver database, as well as conducted RT-qPCR and Western blot analyses. In the CCLE-Liver database, mRNA levels of PHLPP2 exhibited an increase in HCC cell lines with Mut p53 (SNU449, Huh7, and PLC/PRF/5) compared to those with Wt p53 (SK-Hep-1 and HepG2) (Fig. S4A). Subsequently, we performed RT-qPCR and Western blotting to assess PHLPP2 mRNA and protein levels in the HCC cell lines we used. RT-qPCR analysis indicated higher mRNA levels of PHLPP2 in the Mut p53 HCC cell lines (SNU449, Huh7, and PLC/PRF/5) compared to the Wt p53 HCC cell lines (SK-Hep-1 and HepG2) (Fig. S4B). Moreover, analysis of The Cancer Genome Atlas Liver Hepatocellular Carcinoma (TCGA-LIHC) database showed that the PHLPP2 expression was not lower in Mut p53 HCC tissues compared to Wt p53 HCC tissues (Fig. S4C). Western blot analysis revealed similar protein levels of PHLPP2 in the Mut p53 HCC cell lines compared to the Wt p53 HCC cell lines (Fig. S4D). In conclusion, our analysis suggests that the basal expression of PHLPP2 is independent with p53 status in HCC cell lines.

### TRIM22 physically interacts with PHLPP2 and promotes its ubiquitin-mediated degradation

**Fig. 3:**
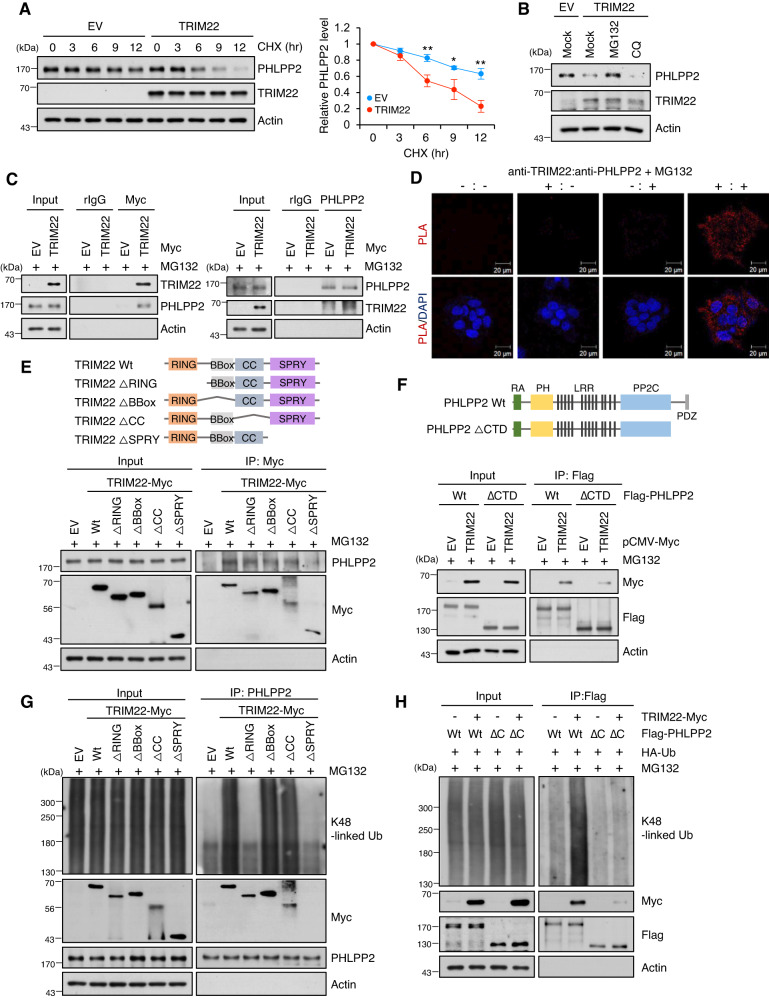
TRIM22 directly binds to PHLPP2 and induces its ubiquitin-mediated degradation. **A** Analysis of PHLPP2 protein stability by Western blotting. TRIM22-overexpressed HepG2 cells were treated with cycloheximide (CHX) for the indicated times, harvested, and analyzed by Western blotting. Data are presented as mean ± SD (unpaired two-tailed *t*-test, **P = 0.0047, *t* = 5.708; *P = 0.0208, *t* = 3.700; **P = 0.0021, *t* = 7.040, *n* = 3). **B** Western blotting analysis of TRIM22-overexpressed HepG2 cells treated with the proteasomal degradation inhibitor, MG132, or the lysosomal inhibitor, CQ. **C** Co-immunoprecipitation (Co-IP) assays for the interaction between TRIM22 and PHLPP2. HepG2 cells were transfected with EV or TRIM22-Myc and then treated with 20 μM MG132 for 4 hrs. The cells were subjected to IP using anti-Myc (left) or anti-PHLPP2 (right) antibodies. Immunoprecipitates were analyzed by Western blotting using the indicated antibodies. **D** Proximity ligation assay (PLA) for cytoplasmic interaction between TRIM22 and PHLPP2 using each antibody. TRIM22-Myc-overexpressing HepG2 cells were treated with 20 μM MG132 for 4 hrs and subjected to PLA. The red spots indicate TRIM22-PHLPP2 interactions. Nuclei were stained with DAPI. Scale bars, 20 μm. **E** Schematic showing the domain structures of TRIM22 Wt and Muts (ΔRING, ΔBBox, ΔCC, or ΔSPRY) (Upper). HepG2 cells were transfected with TRIM22 Wt or Muts (ΔRING, ΔBBox, ΔCC, or ΔSPRY). IP was performed and immunoprecipitates were subjected to Western blot analysis. **F** Schematic showing the domain structures of PHLPP2 Wt and PHLPP2 ΔC-terminal (ΔCTD) (Upper). Flag-tagged PHLPP2 Wt or PHLPP2 ΔCTD was co-transfected with TRIM22 Wt into HepG2 cells. PHLPP2 Wt or PHLPP2 ΔCTD was immunoprecipitated using Flag antibody, and the immunoprecipitates were analyzed using Western blotting (Bottom). **G** Ubiquitination assays of PHLPP2 in TRIM22 Wt or Muts (ΔRING, ΔBBox, ΔCC, or ΔSPRY)-overexpressed HepG2 cells. **H** Co-IP and Western blot analysis of PHLPP2 Wt or PHLPP2 ΔCTD K48 ubiquitination in TRIM22 Wt-overexpressed HepG2 cells.

To examine whether TRIM22 directly regulates the level of PHLPP2, we used cycloheximide (CHX) to block de novo protein synthesis in TRIM22-overexpressed or IR-treated HepG2 cells. We found that TRIM22 overexpression significantly decreased the PHLPP2 protein levels in TRIM22-overexpressed and IR-treated cells (Fig. 3A, Fig. S5A, B). The PHLPP2 protein level in this system was rescued by the proteasome inhibitor, MG132, whereas the lysosome inhibitor, chloroquine (CQ), had no effect on this protein level (Fig. 3B, Fig. S5C). To assess whether TRIM22 regulates the PHLPP2 protein level through a physical association, we performed reciprocal immunoprecipitations (IP) in HepG2 cells transfected with TRIM22-Myc. TRIM22 and PHLPP2 could be reciprocally precipitated using either anti-Myc or anti-PHLPP2 in TRIM22-Myc-transfected cells, indicating that there is a physical interaction between TRIM22 and PHLPP2 (Fig. 3C). The cytoplasmic interaction between TRIM22 and PHLPP2 was further confirmed by the results of a proximity ligation assay (PLA) (Fig. 3D). Moreover, the interaction between TRIM22 and PHLPP2 was increased in IR-induced senescent HepG2 cells (Fig. S5D, E). Together, these findings demonstrate that TRIM22 directly interacts with PHLPP2 and regulates its protein level to induce cellular senescence.

To elucidate the TRIM22 domain responsible for the interaction with PHLPP2, we generated Myc-tagged TRIM22 truncation mutants with deletions in the RING domain (ΔRING), BBox domain (ΔBBox), CC domain (ΔCC) or SPRY domain (ΔSPRY) (Fig. 3E, Upper). Co-IP and Western blot analyses demonstrated that the SPRY domain of TRIM22 was essential for interaction with PHLPP2, while the other domains (RING, BBox, and CC) did not influence the interaction with PHLPP2 (Fig. 3E, Bottom). Substrate phosphorylation can influence the substrate-E3 ligase interaction, leading to ubiquitination and degradation of the substrate [34]. Review of the PhosphoSite database (www.phosphosite.org) revealed that PHLPP2 contains 10 phosphorylation sites at its C-terminal domain. To test whether phosphorylation of PHLPP2 might be responsible for its association with TRIM22, we generated PHLPP2 truncation mutants lacking the C-terminal domain (ΔCTD) (Fig. 3F, Upper). We found that PHLPP2 ΔCTD could not bind to TRIM22 (Fig. 3F, Bottom). This indicates that the SPRY domain of TRIM22 and the C-terminus of PHLPP2 are essential for the interaction between these proteins. Furthermore, TRIM22 Wt, ΔBBox, or ΔCC induced K48-linkage polyubiquitination of PHLPP2, whereas TRIM22 ΔRING or ΔSPRY had no effect on PHLPP2 ubiquitination (Fig. 3G). In IR-induced senescent HepG2 cells, the upregulation of TRIM22 expression increased the K48-linked polyubiquitination levels of PHLPP2, whereas TRIM22 depletion decreased this parameter (Fig. S5F). TRIM22-induced the K48-linked polyubiquitination of PHLPP2 Wt, but not its C-terminal deleted mutant (PHLPP2 ΔCTD), which was validated using a K48-linkage specific polyubiquitin antibody (Fig. 3H). In TRIM22 Wt-overexpressed cells, the PHLPP2 protein level was decreased and AKT-p53-p21 signaling was activated, finally leading to cellular senescence (Fig. S6A–C). However, mutant TRIM22 lacking the RING-finger or SPRY domain failed to regulate the PHLPP2 protein level, activate downstream AKT-p53-p21 signaling, and induce cellular senescence (Fig. S6A–C).

These results demonstrate that TRIM22 physically interacts with PHLPP2 and promotes its ubiquitin-mediated proteasomal degradation, ultimately leading to cellular senescence due to AKT activation in HCC cells.

### Phosphorylation of PHLPP2 mediated by IKKβ promotes the PHLPP2-TRIM222 interaction

**Fig. 4:**
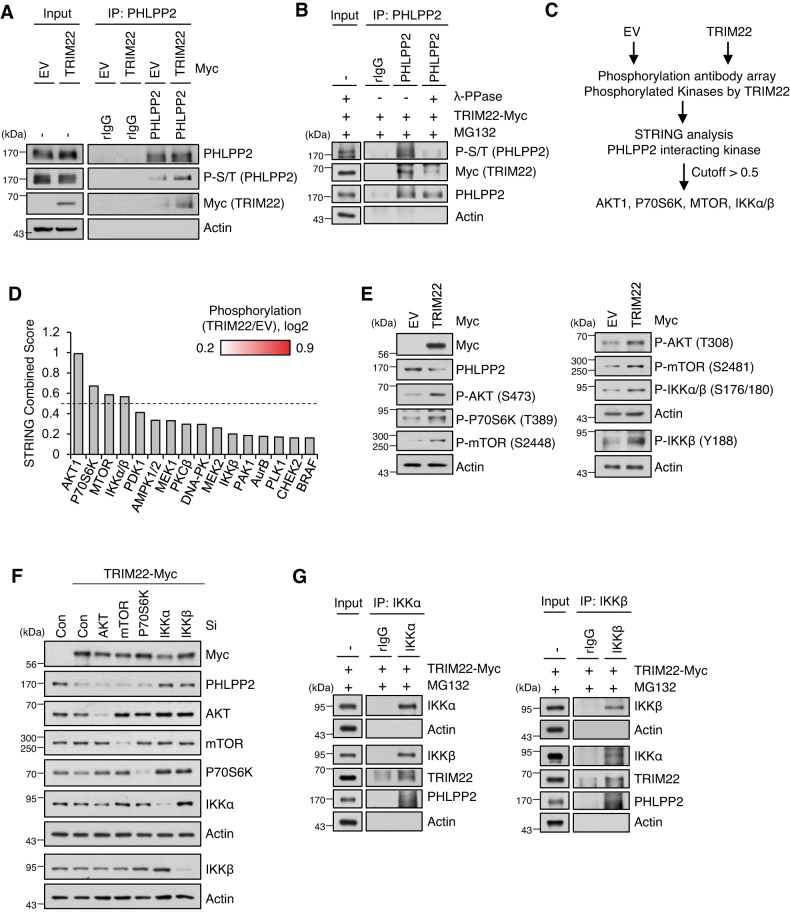
PHLPP2 is regulated by IKKα and IKKβ in TRIM22-overexpressed cells. **A** IP using anti-PHLPP2 in TRIM22-overexpressed HepG2 cells. Immunoprecipitates were analyzed by Western blotting using the indicated antibodies. **B** IP was performed using anti-PHLPP2 antibody in TRIM22-overexpressed HepG2 cells. After IP, lysates were treated with λ-PPase and analyzed by Western blotting using the indicated antibodies. **C** Workflow of strategy used to identify kinase candidates that interact with and phosphorylate PHLPP2. **D** STRING analysis of PHLPP2-interacting kinases. **E** Western blot analysis of kinases in TRIM22-overexpressed HepG2 cells. **F** HepG2 cells were transfected with siRNA targeting each indicated kinase. After 48 hrs, the cells were harvested and analyzed by Western blotting using the indicated antibodies. **G** IP using anti-IKKα (left) or anti-IKKβ (right) in TRIM22-overexpressed HepG2 cells. Immunoprecipitates were analyzed by Western blotting using the indicated antibodies.

Next, we found that the phosphorylation level of PHLPP2 was increased upon TRIM22 overexpression (Fig. 4A). When we treated TRIM22-overexpressed cells with Lambda protein phosphatase (λ-PPase), dephosphorylated PHLPP2 failed to interact with TRIM22 (Fig. 4B), indicating that the binding of PHLPP2 to TRIM22 was occurred though PHLPP2 phosphorylation per se. Thus, we investigated which kinase is responsible for PHLPP2 phosphorylation in TRIM22-overexpressed HCC cells. Our phosphoprotein array analysis revealed that TRIM22 overexpression led to the phosphorylation of 21 kinases (Fig. 4C). Using the STRING software with a confidence cutoff of 0.5, we predicted that AKT1, P70S6K, mTOR, and IKKα/β could potentially interact with PHLPP2 (Fig. 4D). Phosphorylation status of those kinases were confirmed by Western blot analysis of TRIM22-overexpressed cells (Fig. 4E). To further clarify the result, we knock downed AKT1, mTOR, P70S6K, IKKα, and IKKβ, and found that depletion of IKKα or IKKβ restored the PHLPP2 protein level in TRIM22-overexpressed cells (Fig. 4F). Moreover, IKKα and IKKβ are interacted with PHLPP2 in TRIM22-overexpressed cells (Fig. 4G).

**Fig. 5:**
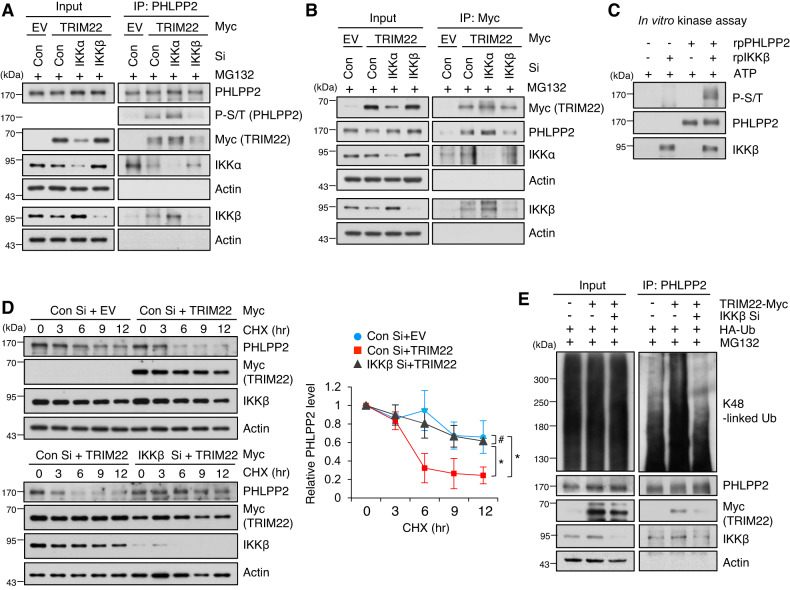
Phosphorylation of PHLPP2 by IKKβ facilitates the interaction between PHLPP2 and TRIM22 and promotes the degradation of PHLPP2 by TRIM22. **A**, **B** IP using anti-PHLPP2 (**A**) or anti-Myc (**B**) was performed in IKKα- or IKKβ-depleted HepG2 cells following TRIM22 overexpression. Immunoprecipitates were analyzed by Western blotting using the indicated antibodies. **C** Western blotting of an in vitro kinase assay performed between IKKβ and PHLPP2. **D** Analysis of PHLPP2 protein stability. Con Si- or IKKβ Si-transfected HepG2 cells were transfected with EV or TRIM22-expressing vector. The cells were treated with cycloheximide (CHX) for the indicated times, harvested, and analyzed by Western blotting. Data are presented as mean ± SD (one-way ANOVA with Tukey’s multiple comparison test, F(14,30) = 14.01, *P = 0.0153; ^#^*P* > 0.9999; *P = 0.0437, *n* = 3). **E** Ubiquitination assay of PHLPP2 in IKKβ-depleted HepG2 cells following TRIM22 overexpression.

To explore the roles of IKKα and/or IKKβ in PHLPP2 phosphorylation, we performed PHLPP2 IP in IKKα- or IKKβ-depleted cells. We found that the interaction between PHLPP2 and TRIM22 was reduced in IKKβ-depleted cells in a PHLPP2 phosphorylation status-dependent fashion, but that this was not seen in IKKα-depleted cells (Fig. 5A). Similar results were obtained from TRIM22 IP assays in IKKα- or IKKβ-depleted cells (Fig. 5B). An in vitro kinase assay revealed that PHLPP2 was directly phosphorylated by IKKβ (Fig. 5C). Consistently, the degradation of PHLPP2 by TRIM22 was decreased in IKKβ-depleted cells (Fig. 5D) and TRIM22-induced K48-linked polyubiquitination of PHLPP2 was reduced by IKKβ knockdown (Fig. 5E). As the phosphorylation of IKKβ was increased in TRIM22-overexpressed HCC cells, we investigated the potential role of TRIM22 in regulating IKKβ phosphorylation. We observed that upregulation of TRIM22 increased the phosphorylation of IKKβ at Y188 in HCC cells with Wt p53, but not Mut p53 (Fig. S7A). Additionally, knockdown of TRIM22 in IR-treated HepG2 cells did not alter the phosphorylation level of IKKβ (Fig. S7B). Furthermore, TRIM22 overexpression in HepG2 cells dose-dependently induced the phosphorylation of IKKβ at Y188, in correlation with PHLPP2 degradation and AKT-p53 signaling activation (Fig. S7C).

In summary, our results indicate that TRIM22 induces the IKKβ-mediated phosphorylation of PHLPP2 and the subsequent degradation of PHLPP2 via ubiquitin-mediated proteasomal degradation, and that this involves a direct interaction between TRIM22 and PHLPP2.

### TRIM22 expression is inversely correlated with PHLPP2 expression in HCC databases and patient specimens

**Fig. 6:**
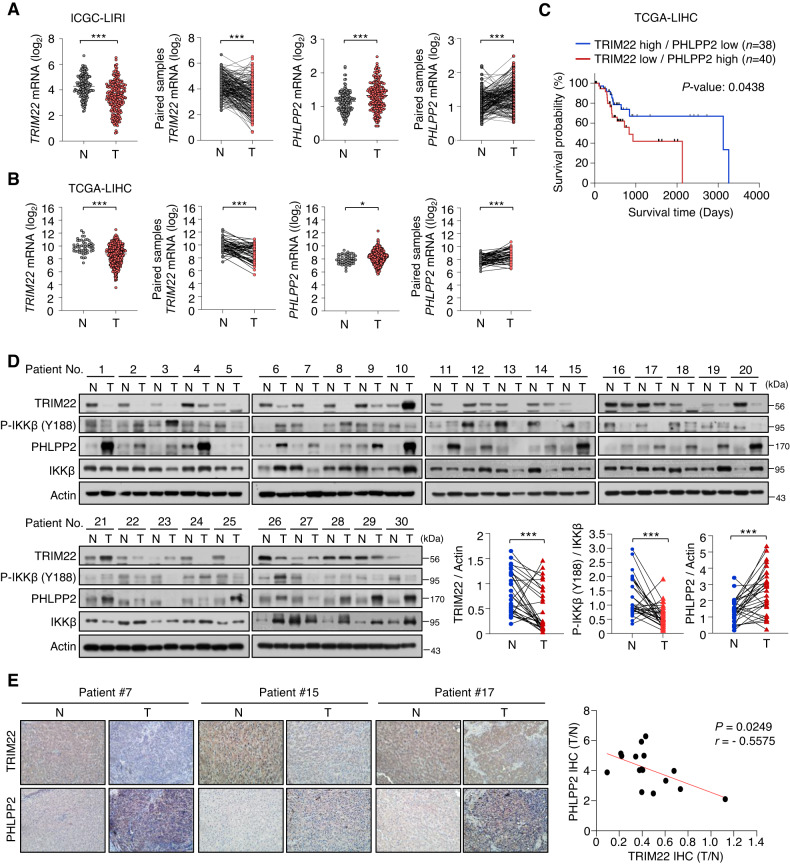
TRIM22 and PHLPP2 levels are inversely correlated in HCC and paired normal patient tissues. **A**, **B** Comparison of TRIM22 and PHLPP2 mRNA levels in HCC and paired normal tissue samples from ICGC-LIRI database [Normal (N), *n* = 177; Tumor (T), *n* = 212. Paired samples, *n* = 177 (**A**)] (TRIM22 mRNA: unpaired two-tailed *t*-test, ***P < 0.0001, *t* = 8.059; paired two-tailed *t*-test, ***P < 0.0001, *t* = 9.223; PHLPP2 mRNA: unpaired two-tailed *t*-test, ***P < 0.0001, *t* = 4.756; paired two-tailed *t*-tes*t*, ***P < 0.0001, *t* = 5.125) and TCGA-LIHC database [N, *n* = 50; T, *n* = 369. Paired samples, *n* = 50 (**B**)] (TRIM22 mRNA: unpaired two-tailed *t*-test, ***P < 0.0001, *t* = 5.485; paired two-tailed *t*-test, ***P < 0.0001, *t* = 5.693; PHLPP2 mRNA: unpaired two-*t*ailed *t*-test, *P = 0.0105, *t* = 2.572; paired two-tailed *t*-test, ***P < 0.0001, *t* = 4.938). **C** Survival analysis between groups with different levels of TRIM22 and PHLPP2 in the TCGA-LIHC database. **D** Western blot analysis of TRIM22, P-IKKβ (Y188), and PHLPP2 in HCC and paired normal patient tissues. *n* = 30. Statistical analysis of the value intensity of TRIM22, PHLPP2, and P-IKKβ (Y188) normalized to those of Actin and Total IKKβ in patient tissues (TRIM22: paired two-tailed *t*-test, ***P = 0.0002, *t* = 4.206; P-IKKβ: paired two-tailed *t-*test, ***P = 0.0004, *t* = 4.003; PHL*P*P2: paired two-tailed *t*-test, ***P = 0.0002, *t* = 4.327). The protein levels were quantified by densitome*t*ry using the ImageJ software and normalized to the protein level of Actin or Total IKKβ. **E** IHC analysis of TRIM22 and PHLPP2 in HCC and paired patient tissues (left). *n* = 16. Scale bars, 50 μm. Correlation of TRIM22 and PHLPP2 IHC scores (right). *r* is the Spearman’s rank correlation coefficient.

To investigate the clinical significance of TRIM22 and PHLPP2 in HCC, we analyzed their expression levels in patients with HCC, as deposited to the International Cancer Genome Consortium Liver Cancer-RIKEN Japan (ICGC-LIRI-JP) (Fig. 6A) and TCGA-LIHC (Fig. 6B) databases. Analyses of these databases revealed that TRIM22 expression was downregulated in both total and paired HCC tumor tissues compared to normal tissues, while PHLPP2 expression was upregulated in HCC tumor tissues (Fig. 6A,B). Furthermore, our TCGA-LIHC database analysis showed that patients with high TRIM22 expression and low PHLPP2 expression had better overall survival (OS) rates (Fig. 6C). To support these findings, we collected 30 pairs of HCC and normal tissues and evaluated the protein levels of TRIM22 and PHLPP2. The results showed that TRIM22 protein levels were lower and PHLPP2 protein levels were higher in HCC tissues compared to normal tissues. Moreover, we observed that levels of phospho-IKKβ (Y188) in HCC tissues were lower than those in normal tissues (Fig. 6D). Furthermore, the IHC scores were negatively correlated between TRIM22 and PHLPP2 in both normal and tumor tissues (Fig. 6E). Taken together, our findings suggest that the expression levels of TRIM22 and PHLPP2 are inversely associated in HCC patient tissues.

## Discussion

The present study demonstrates that TRIM22 promotes HCC senescence by activating the AKT-p53-p21 signaling pathway. AKT, a serine and threonine kinase, is activated by phosphorylation at T308 or S473, and regulates cell survival, proliferation, growth, and glycogen metabolism [35, 36]. Our group and others reported that, although AKT is associated with tumor initiation and progression, its activation can induce cellular senescence in both non-transformed and cancer cells [37–41]. This AKT-induced cellular senescence (AIS) can be triggered by various conditions, such as overexpression of myristoylated-AKT (Myr-AKT) and activation of receptors upstream of AKT [39]. AIS can serve as a fail-safe mechanism against tumorigenesis. Our group and others also demonstrated that the loss of phosphatase and tensin homolog (PTEN), a major negative regulator of PI3K/AKT signaling, can induce cellular senescence in a p53-dependent manner that is called PTEN-induced cellular senescence (PICS) [37–39, 42]. These findings emphasize that AKT plays dual roles in critically regulating cell fate decisions between proliferation and senescence, depending on cellular context.

The induction of cellular senescence is an important anticancer strategy, as it can suppress tumor growth and enhance the vulnerability to combination treatments. Senescence can be triggered by various cancer therapies, particularly IR treatment. To identify E3 ligases that are involved in regulating therapy-induced senescence, we herein conducted expression profiling analysis of IR-treated senescent HepG2 cells. We found that TRIM22 is upregulated in response to IR-exposure in HCC cells. TRIM22 is an E3 ligase that can exhibit both tumor-promoting and tumor-suppressive roles in different cancers. Several TRIM22 isoforms play crucial roles in cancer biology. For instance, TRIM47 has been implicated in promoting tumor progression in colon and pancreatic cancer by degrading SMAD4 and FBP1 [43, 44]. In HCC, TRIM25 enhances tumor cell survival by targeting Keap1 for degradation [20]. Conversely, TRIM7 and TRIM50 have been reported to suppress HCC progression by directly targeting Src and SNAIL for degradation, respectively [45, 46]. In colorectal cancer, TRIM67 functions as tumor suppressor by inducing p53-induced apoptosis and inhibiting cell growth [47]. TRIM22 is upregulated in glioblastoma (GBM) and promotes tumor growth and progression by modulating the stability of IKKγ and IkBα [29]. Conversely, TRIM22 is downregulated in osteosarcoma (OS) and gastric cancer [30, 31]. Overexpression of TRIM22 suppresses the proliferation and metastasis of OS cells by targeting NRF2 for degradation and activating the ROS/AMPK/mTOR/autophagy signaling pathway [31]. In gastric cancer cells, TRIM22 inhibits cancer cell proliferation and migration by reducing the phosphorylation of SMAD2 [30]. The present study demonstrates that TRIM22 critically contributes to the therapy-induced senescence of HCC cells. Our data indicated that TRIM22 functions as a tumor suppressor by directly regulating the level of PHLPP2. Previous studies have reported that TRIM22 induces apoptosis in osteosarcoma, monocyte, and neuron cells [31, 48, 49]. However, in this study, we revealed that TRIM22 is a critical factor in cellular senescence in HCC cells. TRIM22 overexpression was shown to suppress cell proliferation by inducing cellular senescence without causing cell death in HCC. Our study showed consistent results from IR-induced and TRIM22-overexpressed senescent HCC cells, and from HCC patient tissues.

**Fig. 7:**
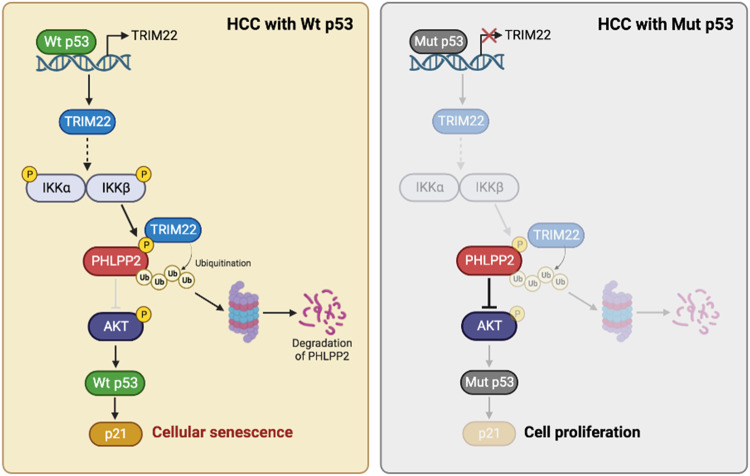
Schematic model proposed according to the findings of the present study. A proposed model for the function of TRIM22 in the degradation of PHLPP2 and the induction of cellular senescence in HCC cells (created with BioRender.com).

p53 is a transcription factor that plays crucial roles in suppressing tumor growth by promoting cellular senescence, apoptosis, DNA repair, and other important processes. Mutations in p53 that result in the loss of its transcriptional activity can lead to cells taking on oncogenic functions, chemo-resistance, and other aspects of tumorigenesis [2, 50, 51]. Approximately 70% of HCC patients having Wt p53 indicates that the frequency of p53 mutations is relatively low in HCC compared to other types of human cancer [50, 51]. The present study demonstrated that TRIM22, which is induced by Wt p53 under the IR-exposed condition, triggers HCC cell senescence by activating the AKT-p53-p21 signaling pathway. These findings indicate that Wt p53 is essential both upstream and downstream of TRIM22 for the induction of HCC cell senescence (Fig. 7). Mechanistically, TRIM22 degrades the AKT phosphatase, PHLPP2, to increase AKT phosphorylation in HCC cells. PHLPP2 belongs to the Pleckstrin Homology Domain Leucine-Rich Repeat Protein Phosphatase (PHLPP) family, the members of which negatively regulate PI3K/AKT signaling by dephosphorylating AKT at T308 and S473 [32]. Moreover, Tantai et al. previously reported that TRIM46 activates AKT signaling by promoting the ubiquitination of PHLPP2 in lung adenocarcinoma (LUAD) [52]. In this study, we found that TRIM22 overexpression phosphorylates PHLPP2, and this phosphorylation is crucial for the interaction of PHLPP2 with TRIM22. Unlike PHLPP2, we evidenced that the other PHLPP family isoform, PHLPP1, failed to mediate senescence in this study. It is reported that TRIM22 overexpression increases the phosphorylation of IKKβ at S181 and Y188, resulting in IKKβ activation [53]. Activated IKKβ induces the phosphorylation and subsequent degradation of substrates by recruiting E3 ligases [54, 55]. Consistent with these previous reports, we observed that PHLPP2 was phosphorylated by IKKβ activation in TRIM22-overexpressed cells, which is crucial for the recruitment of TRIM22. Furthermore, TRIM22 and IKKβ were negatively correlated with PHLPP2 in HCC patient samples.

Conclusively, we reveal a novel mechanism in which TRIM22 regulates PHLPP2 to promote HCC senescence. Targeting TRIM22 may present a promising therapeutic approach for the treatment of cancers, offering a new avenue for intervention in cancer therapy.

## Materials and methods

### Cell lines

HCC cell lines HepG2, SK-Hep-1, SNU449, and PLC/PRF/5 were purchased from the ATCC. HepG2 and PLC/PRF/5 cells were cultured in Minimum Essential Medium Eagle (MEM; WelGene, Daegu, Korea). SK-Hep-1 cells were cultured in Dulbecco’s Modified Eagle Medium (DMEM; WelGene). SNU449 cells were cultured in Roswell Park Memorial Institute Medium 1640 (RPMI-1640; WelGene). All the cell lines, supplemented with 10% fetal bovine serum (FBS; Gibco, Grand Island, New York, USA) and 1% penicillin/streptomycin (WelGene) were incubated at 37 °C in a 5% CO_2_ incubator.

### Transfection of siRNA and plasmids

Transfection of siRNAs (Bioneer, Daejeon Korea) and plasmids were performed using RNAi-MAX (Invitrogen, Carlsbad, CA, USA) and Lipofectamine 2000 (Invitrogen). Transfection medium were exchanged by regular growth media 6 hrs after transfection. The sequences of siRNAs used in this study were listed in Table S1.

### Plasmid constructs

Full-length TRIM22 or TRIM22 mutants (TRIM22 ΔRING, ΔBBox, ΔCC or ΔSPRY) were obtained from pCMV6-TRIM22-Myc-DDK (RC207431, Origene, Rockville, MD, USA) and cloned into the pCMV-Myc vector. Full-length PHLPP2 was provided by Dr. KyeongJin Kim at Inha University, Korea and cloned into the p3xFlag vector. PHLPP2 ΔC-terminal (CTD) mutant were prepared based on p3xFlag-PHLPP2 Wt. All gene fragments were obtained by PCR amplification. The primers for plasmid construction used in this study were listed in Table S2.

### Proximity ligation assay (PLA)

HepG2 cells seeded onto the glass coverslips were transfected with indicated plasmids or treated with IR. After 48 hrs, the cells were treated with 20 μM MG132 for 4 hrs. Cells were fixed with 4% paraformaldehyde for 10 min at room temperature, permeabilized with 0.1% Triton X-100 for 10 min and washed with DPBS. Protein-protein interactions were detected using Duolink^®^ PLA kit (Sigma–Aldrich, St Louis, MO, USA) according to the manufacturer’s instructions. The coverslips were mounted using Duolink^®^ In Situ Mounting Medium with DAPI (Sigma–Aldrich). Immunofluorescence was detected and visualized using Zeiss LSM510 confocal microscope.

### RNA extract, reverse transcription and quantitative PCR (RT-qPCR)

Total RNA was extracted from cells and tissues using TRIzol reagent (Molecular Research Center, Netherlands). RNA was reverse transcribed to cDNA using M-MLV reverse transcriptase (Thermo Fisher Scientific, Waltham, MA, USA) according to the manufacturer’s instruction, and qPCR was performed using iQTM SYBR^®^ Green Supermix (BioRad Laboratories, Hercules, CA, USA) in a CFX ConnectTM RT-PCR Detection System (BioRad Laboratories). Housekeeping gene Actin was used as internal controls to normalize target mRNAs. The primer sequences for RT-qPCR were listed in Table S3.

### Immunoprecipitation (IP) and Chromatin Immunoprecipitation (ChIP)

For IP, cells were lysed in NET-2 buffer (1 M Tris–HCl (pH 7.4), 5 M NaCl, 10% NP-40, 0.1 M PMSF and 0.2 M Benzamidine) containing 2.5 mM sodium pyrophosphate, 1 mM β-glycerophosphate and 1 mM sodium orthovanadate. To remove non-specific bindings, cell lysates were pre-cleared using either protein A-Sepharose beads (GE Healthcare Bio-Science AB, Uppsala, Sweden) or G-Resin (GenScript, Piscataway, NJ, USA). Pre-cleared lysates were immunoprecipitated with specific antibodies or IgG for 4 hrs, followed by overnight incubation with protein A/G beads. The immunoprecipitants were washed with NET-2 buffer and eluted with 2× Laemmli sample buffer, followed by incubation at 95 °C for 10 min. For ChIP, IR-treated HepG2 cells were cross-linked with 1% formaldehyde for 5 min and stopped by 0.125 M glycine for 10 min at room temperature. The cells were washed with cold PBS, lysed with NET-2 buffer, and pre-cleared with protein A-Sepharose beads. The lysates were immunoprecipitated with either anti-p53 (Santa Cruz, FL-393) or rabbit IgG for 4 hrs, followed by incubation with protein A beads overnight. After washing with NET-2 buffer, DNAs bounds to proteins were purified by phenol-chloroform extraction and precipitated in ethanol. The DNA was then resuspended in DNase-and RNase-free water and analyzed by qPCR analysis. The proteins were detected using Western blot analysis.

### Western blotting

The cells were lysed in RIPA buffer (50 mM Tris–HCl pH 8.0, 150 mM NaCl, 1% NP-40, 2 mM EDTA, 0.1% SDS, 0.5% sodium deoxycholate) containing protease inhibitor (Roche) and phosphatase inhibitor (Sigma–Aldrich). The protein concentration of whole cell lysates was determined using Bradford Protein Assay (BioRad Laboratories). Equal amounts of proteins were mixed with 2× Laemmli sample buffer and incubated for 5 min at 95 °C. The proteins were separated by SDS-PAGE, transferred to a nitrocellulose membrane (GE Healthcare, Buckinghamshire, UK), and blocked with 5% Bovine Serum Albumin (BSA). The membranes were then incubated with the primary antibodies for overnight at 4 °C. After washing with 1× TBST, the membranes were incubated with an HRP-linked secondary antibody and signals were detected using Pierce™ ECL Western Blotting Substrate (Thermo Fisher Scientific). Full and uncropped Western blot images have been uploaded in Supplemental Material.

### Caspase-3 activity

Caspse-3 activity was analyzed with NucView 488 Caspase-3 (10403, BIOTIUM, San Francisco, CA, USA) according to the manufacturer’s instruction. Briefly, cells were seeded in 60 mm dishes, cultured for 24 hrs, and transfected with indicated plasmids or treated with IR. After 48 hrs, the cells were replaced medium with PBS containing 5 μM NucView® 488 substrate stock solution and incubated for 30 min at room temperature. Next, the cells were incubated with Hoechst (Invitrogen) for 10 min at room temperature. After incubation, the cells were washed with PBS and observed by using Olympus CKX41 light microscope (Olympus, Tokyo, Japan). Activity of caspase-3 was analyzed by the ImageJ software (version 1.52).

### Reagents and antibodies

Reagents were obtained from the following suppliers: Doxorubicin (D1515, Sigma–Aldrich), MG132 (C2211, Sigma–Aldrich), Cycloheximide (CHX) (C7698, Sigma–Aldrich), Chloroquine (CQ) (C6628, Sigma–Aldrich), Sodium chloride (NaCl) (7647-14-5, Duchefa), Magnesium chloride (MgCl_2_) (M2670, Sigma–Aldrich), Citric acid (C1909, Sigma–Aldrich), Sodium phosphate (S3264, Sigma–Aldrich), Potassium ferrocyanide (P9387, Sigma–Aldrich), Potassium ferricyanide (P8131, Sigma–Aldrich), X-galactosidase (X-Gal) (7002, Beamsbio), Tris (T1801, Duchefa), β-glycerophosphate (G9422, Sigma–Aldrich), Dithiothreitol (DTT) (P2325, Invitrogen), Sodium orthovanadate (Na_3_VO_4_) (567540, Sigma–Aldrich), ATP (P0756, NEB), NP-40 (68987-90-6, USB), Ethylenediaminetetraacetic acid (EDTA) (03609, Sigma–Aldrich), Sodium dodecyl sulfate (SDS) (3771, Sigma–Aldrich), Sodium deoxycholate (D6750, Sigma–Aldrich), Phenylmethanesulfonylfluoride fluoride (PMSF) (52332, Millipore), Benzamidine (434760, Sigma–Aldrich), Sodium pyrophosphate (71501, Sigma–Aldrich), Sodium Fluoride (NaF) (201154, Sigma–Aldrich).

Antibodies were obtained from the following suppliers: Myc-Taq (9B11) (2276, CST), p53 (DO7) (NCL-L-p53-DO7, Leica), p21 Waf1/Cip1 (12D1) (2947, CST), TRIM22 (ab224059, abcam), β-Actin (8H10D10) (3700, CST), P-AKT S473 (D9E) (4060, CST), P-AKT T308 (D25E6) (13038, CST), P-mTOR S2448 (D9C2) (5536, CST), P-mTOR S2481 (2974, CST), AKT (9272, CST), PHLPP1 (A300-660A, Bethyl), PHLPP2 (A300-661A, Bethyl), PHLPP2 (NBP2-13757, Novus), C-PARP (CST), Flag-M2 peroxidase (A8592, Sigma–Aldrich), HA-Tag (C29F4) (3724, CST), P-(Ser/Thr) Phe (ab17464, abcam), P-P70S6K T389 (9206, CST), P-IKKα/β S176/S180 (16A6) (2697, CST), P70S6K (9202, CST), IKKα (EPR464) (ab109749, abcam), IKKβ (D30C6) (8943, CST), P-IKKβ Y188 (bs-3233, Bioss).

### Senescence-associated β-galactosidase (SA-β-Gal) staining

To evaluate SA-β-Gal activity, cells were stained with the method described previously [56]. Briefly, cells were washed with DPBS and fixed with 3.7% formaldehyde for 5 min at room temperature. Fixed cells were incubated for 16 hrs with 1 mL staining solution (150 mM NaCl, 2 mM MgCl_2_, 40 mM citric acid/sodium phosphate pH 6.0, 5 mM potassium ferrocyanide, 5 mM potassium ferricyanide 1 mg/ml X-galactosidase). Images were acquired by Olympus CKX41 using TOMORO AcquPRO 2005.

### Phosphoprotein antibody array

The Phospho Explorer Antibody Assay (Cat# PEX100, Fullmoon Biosystems, Sunnyvale, CA, USA) was used to measure phosphorylation status in EV- or TRIM22-transfected cells. This array was consisted of 1318 antibodies associated with various signaling pathways. Transfected cells were lysed with non-denaturing lysis buffer. Extracted proteins were biotinylated and then incubated on antibody array slides. After incubation, signals of proteins were detected by dye-labeled streptavidin. Raw signal intensity was normalized to all signals on the array slides. Fold changes between samples were calculated and proteins with fold changes above 1.2-fold and below 0.8-fold were included in the final dataset. Array experiments and analysis were performed as services by Ebiogen lnc.

### Cell viability and Edu assay

Cultured cells were treated with Trypsin-EDTA and collected in order to assess cell viability. Suspended cells were diluted 1:1 with 0.4% (w/v) trypan blue solution (Gibco) and counted using a hemocytometer. To determine cell proliferation, cells were labeled with EdU Staining Proliferation Kit (iFluor488) (Abcam, ab219801) according to the manufacturer’s instruction. Briefly, cells were seeded on glass coverslips and transfected with EV or TRIM22. After 4 days, cells were incubated with the cell culture medium containing 20 μM EdU for 2 hrs at 37 °C incubator. After fixation and permeabilization, cells were stained with iFluor488 azide and Hoechst. Cells were visualized by using a fluorescent microscope Olympus IX83. Edu positivity was analyzed by the ImageJ software (version 1.52).

### In vitro kinase assay

Recombinant proteins of PHLPP2-Myc/Flag and GST-IKKβ (Active) were supplied by Origene and SignalChem, respectively. For phosphorylation reaction, 0.5 μg PHLPP2-Myc/Flag was incubated with 0.2 μg GST-IKKβ (Active) in 20 μl kinase reaction buffer (25 mM Tris–HCl (pH 7.5), 5 mM β-glycerophosphate, 2 mM DTT, 0.1 mM Na_3_VO_4_, 10 mM MgCl_2_, 200 μM ATP) at 30 °C for 30 min. Kinase reaction was stopped by adding 2× Laemmli sample buffer and incubation for 10 min at 95 °C. Phosphorylation of PHLPP2 was analyzed by Western blotting.

### Human HCC tissue samples

The biospecimens and data of patients with HCC used in this study were provided by the Biobank of InJe University Paik Hospital (InjeBiobank) and Keimyung University Dongsan Hospital Biobank, member of the Korea Biobank Network. This study was performed with the approval of institutional review board (IRB) of Inha University (IRB no. 210408-1AR).

### Immunohistochemistry

Paraffin-embedded tissues were deparaffinized in xylene and rehydrated with a grade series of ethanol solution. Tissue immunostaining was performed using Rabbit specific HRP/DAB detection IHC Kit (abcam, ab64261) according to the manufacturer’s instruction. Antigen retrieval was performed with citrate buffer (pH 6.0) in a microwave. Tissue slides were blocked with hydrogen peroxide block and incubated with primary antibodies against TRIM22 (1:500 dilution, abcam, ab224059) and PHLPP2 (1:100 dilution, Novus, NBP2-13757) overnight at 4 °C. The slides were then incubated with biotinylated goat anti rabbit IgG(H + L) and streptavidin peroxidase for 10 min at room temperature, respectively. The slides were stained with diaminobenzidine (DAB) and then counterstained with hematoxylin. We used FIJI to quantify and analyze IHC scores of TRIM22 and PHLPP2.

### ICGC, TCGA, and CCLE database analysis

RNA-Seq data and clinical information of HCC patients were collected from JP Project from International Cancer Genome Consortium (ICGC-LIRI-JP) and The Cancer Genome Atlas (TCGA) database, and these datasets were extracted from Database of Hepatocellular Carcinoma Expression Atlas (HCCDB) (http://lifeome.net/database/hccdb) and Broad Institute GDAD Firehorse (https://gdac.broadinstitute.org/). TCGA survival data was obtained from OncoLnc (http://www.oncolnc.org). For the gene expression analysis in cancer cell lines, we downloaded the expression data from Cancer Cell Line Encyclopedia (CCLE) (https://portals.broadinstitute.org/ccle).

### Quantification and statistical analysis

All statistical analyses were performed using GraphPad Prism 9 software (version 9.2.0). Student’s *t*-test and one-way ANOVA test were employed, followed by Tukey’s post hoc analysis to determine the significance levels. Pearson’s correlation coefficient was used to assess correlations between gene expressions. Results are presented as the means ± SD from three independent experiments. *P* > 0.05 (#: non-significant); *P* < 0.05 was considered statistically significant; *P < 0.05, **P < 0.01, and ***P < 0.001.

### Reporting summary

Further information on research design is available in the Nature Research Reporting Summary linked to this article.

### Supplementary information

Supplementary Figure Legends

Supplementary Figures

Original Western Blots

Supplementary Table S1

Supplementary Table S2

Supplementary Table S3

Reporting summary

## Data Availability

All experimental datasets generated and analyzed during the current study are included in this published article and its supplementary information files. Additional data and further information are available from the corresponding author upon reasonable request.
