# Supplementary material for: TRIM22 induces cellular senescence by targeting PHLPP2 in hepatocellular carcinoma
Source: Cell Death Dis. 2024 Jan 10;15(1):26. doi: 10.1038/s41419-024-06427-w (PMC10781680; doi:10.1038/s41419-024-06427-w)
Supplement: Supplementary file 1 — Supplementary Figure Legends [file 41419_2024_6427_MOESM1_ESM.docx]

**SUPPLEMENTARY FIGURE LEGENDS**

**Fig. S1 TRIM22 is upregulated by p53 in IR-treated HCC cells**. **A** TRIM22 expression levels in HCC cells with Wt p53 (SK-Hep-1 and HepG2) or Mut p53 (SNU449, Huh7 and PLC/PRF/5) after IR treatment. **B-D** HCC cell lines with Wt p53 (SK-Hep-1 and HepG2) were exposed to IR (8 or 16 Gy). **B** Western blot analysis was performed in HCC cell lines, using the indicated antibodies. **C, D** Cell death was determined by analyzing the activity of caspase-3. Nuclei were stained with Hoechst. Scale bars, 50 μm (**C**) and the level of caspase-3 activity was quantified using ImageJ (**D**). Data are represented as mean ±SD (one-way ANOVA with Tukey’s multiple comparison test, SK-Hep-1: F(3,8)=101.1, #*P*=0.9408, #*P*=0.9902, *n*=3; HepG2: F(3,8)=242.3, #*P*=0.9999, #*P*=0.9704, *n*=3). Positive control (PC) for dead cells, Doxorubicin 2 μg/mL. **E** Analysis of TRIM22 expression in Wt p53 HCC (*n* = 254) and Mut p53 HCC (*n* = 113) from the TCGA-LIHC database (unpaired two-tailed *t* test, ****P*<0.0001, *t*=6.311). **F** HepG2 cells were transfected with Control siRNA (Con Si) or p53 Si, followed by IR treatment. Western blotting (left) and RT-qPCR (right) were performed. Data are presented as mean ±SD (one-way ANOVA with Tukey’s multiple comparison test, F(3,8)=67.93, ****P*<0.0001; ****P*<0.0001, *n*=3). **G** ChIP-qPCR analysis for the enrichment of p53 at its response element in intron 1 of TRIM22. Data are presented as mean ±SD (one-way ANOVA with Tukey’s multiple comparison test, F(3,8)=65.82, ***P*=0.0011, *n*=3).

**Fig. S2 TRIM22 overexpression does not induce cell death in HCC cell lines.** **A-F** TRIM22 is overexpressed in HCC cells with Wt p53 (HepG2 and SK-Hep-1) or Mut p53 (SNU449). **A**, **C**, **E** Western blot analysis of HCC cell lines was performed using the indicated antibodies. **B**, **D**, **F** Cell death was determined by analyzing the activity of caspase-3. Nuclei were stained with Hoechst. Scale bars, 50 μm. Positive control (PC) for dead cells, Doxorubicin 2 μg/mL. Data are presented as mean ±SD (unpaired two-tailed *t* test, B: #*P*=0.2627, *t*=1.303, *n*=3; D: #*P*=0.2437, *t*=1.336, *n*=3; F: #*P*=0.3689, *t*=1.012, *n*=3).

**Fig. S3 Depletion of PHLPP2 induces cellular senescence through the AKT-p53-p21 pathway.** **A-C** HepG2 cells were transfected with each siRNA against PHLPP1 or PHLPP2. Western blot (**A**), cell counting (**B**) Data are presented as mean ±SD (one-way ANOVA with Tukey’s multiple comparison test, F(4,10)=21.29, #*P*=0.1599; #*P*=0.3691; ****P*=0.0002; ****P*=0.0003, *n*=3), Positive control (PC) for dead cells, Doxorubicin 2 μg/mL and SA-β-Gal assays (**C**) Data are presented as mean ±SD (one-way ANOVA with Tukey’s multiple comparison test, F(4,10)=38.26, #*P*>0.9999; #*P*>0.9999; ****P*<0.0001; ****P*=0.0001, *n*=3) were conducted.

**Fig. S4 Expression of PHLPP2 is independent with p53 status. A** PHLPP2 mRNA levels in HCC cell lines with Wt p53 or Mut p53 from the CCLE-Liver database. **B** Relative PHLPP2 mRNA levels in HCC cell lines with Wt p53 or Mut p53 measured by RT-qPCR. Data are presented as mean ±SD (unpaired two-tailed *t* test, ***P*=0.0016, *t*=7.585; **P*=0.0106, *t*=4.530; ***P*=0.0051, *t*=5.560; **P*=0.253, *t*=3.481, *n*=3) **C** Analysis of PHLPP2 expression in Wt p53 HCC (*n* = 254) and Mut p53 HCC (*n* = 113) from the TCGA-LIHC database (unpaired two-tailed *t* test, #*P*=0.4814, *t*=0.705). **D** PHLPP2 protein levels in HCC cell lines with Wt p53 or Mut p53 were measured by Western blot (left) and Western blot results were quantified (right). Data are presented as mean ±SD (unpaired two-tailed *t* test, #*P*=0.0960, *t*=2.168; ****P*=0.0002, *t*=12.76; #*P*=0.0835, *t*=2.294; #*P*=0.7247, *t*=0.3780, *n*=3).

**Fig. S5 IR-upregulated TRIM22 promotes the ubiquitin-mediated degradation of PHLPP2 in HCC cells.** **A** Western blotting was performed in IR-treated HepG2 cells. **B** Analysis of PHLPP2 protein stability. Con Si- or TRIM22 Si-transfected HepG2 cells were exposed to IR and then treated with cycloheximide (CHX) for the indicated times. Data are presented as mean ±SD (one-way ANOVA with Tukey’s multiple comparison test, F(11,24)=24.09, ***P*=0.0062; #*P*=0.9870; ****P*=0.0004, *n*=3). **C** Western blotting analysis of IR-induced senescent HepG2 cells treated with the proteasomal degradation inhibitor, MG132, or the lysosomal inhibitor, CQ. **D** IP using anti-TRIM22 (left) or anti-PHLPP2 (right) antibodies in IR-induced senescent HepG2 cells. **E** PLA for interaction between TRIM22 and PHLPP2 in senescent HepG2 cells, performed using each antibody. IR-induced senescent HepG2 cells were treated with 20 μM MG132 for 4 hrs and subjected to PLA. The red spots indicate TRIM22-PHLPP2 interactions. Nuclei were stained with DAPI. Scale bars, 20 μm. **F** Ubiquitination assays of PHLPP2 in IR-treated HepG2 cells after Con Si or TRIM22 Si transfection.

**Fig. S6 RING and SPRY domains of TRIM22 are critical for its ability to mediate PHLPP2 degradation and cellular senescence in HCC cells.** **A-C** HepG2 cells were transfected with TRIM22 Wt, TRIM22 ΔRING, or TRIM22 ΔSPRY. Western blotting (**A**), relative cell number (**B**) Data are presented as mean ±SD (one-way ANOVA with Tukey’s multiple comparison test, F(3,8)=48.45, ****P*<0.0001; #*P*=0.8313; #*P*=0.8976, *n*=3), Positive control (PC) for dead cells, Doxorubicin 2 μg/mL and SA-β-Gal positivity (**C**) Data are presented as mean ±SD (one-way ANOVA with Tukey’s multiple comparison test, F(3,8)=57.11, ****P*<0.0001; #*P*=0.8601; #*P*=0.9981, *n*=3) were analyzed.

**Fig. S7 Upregulation of TRIM22 increases the phosphorylation of IKKβ.** **A** Western blotting was performed in HCC cell lines with Wt p53 or Mut p53. **B** Western blotting was performed in TRIM22-depleted HepG2 cells after IR treatment. **C** Western blotting was performed in HepG2 cells transfected with TRIM22.
