# Supplementary material for: TRIM22 induces cellular senescence by targeting PHLPP2 in hepatocellular carcinoma
Source: Cell Death Dis. 2024 Jan 10;15(1):26. doi: 10.1038/s41419-024-06427-w (PMC10781680; doi:10.1038/s41419-024-06427-w)
Supplement: Supplementary file 2 — Supplementary Figures [file 41419_2024_6427_MOESM2_ESM.pptx]

## Slide 1
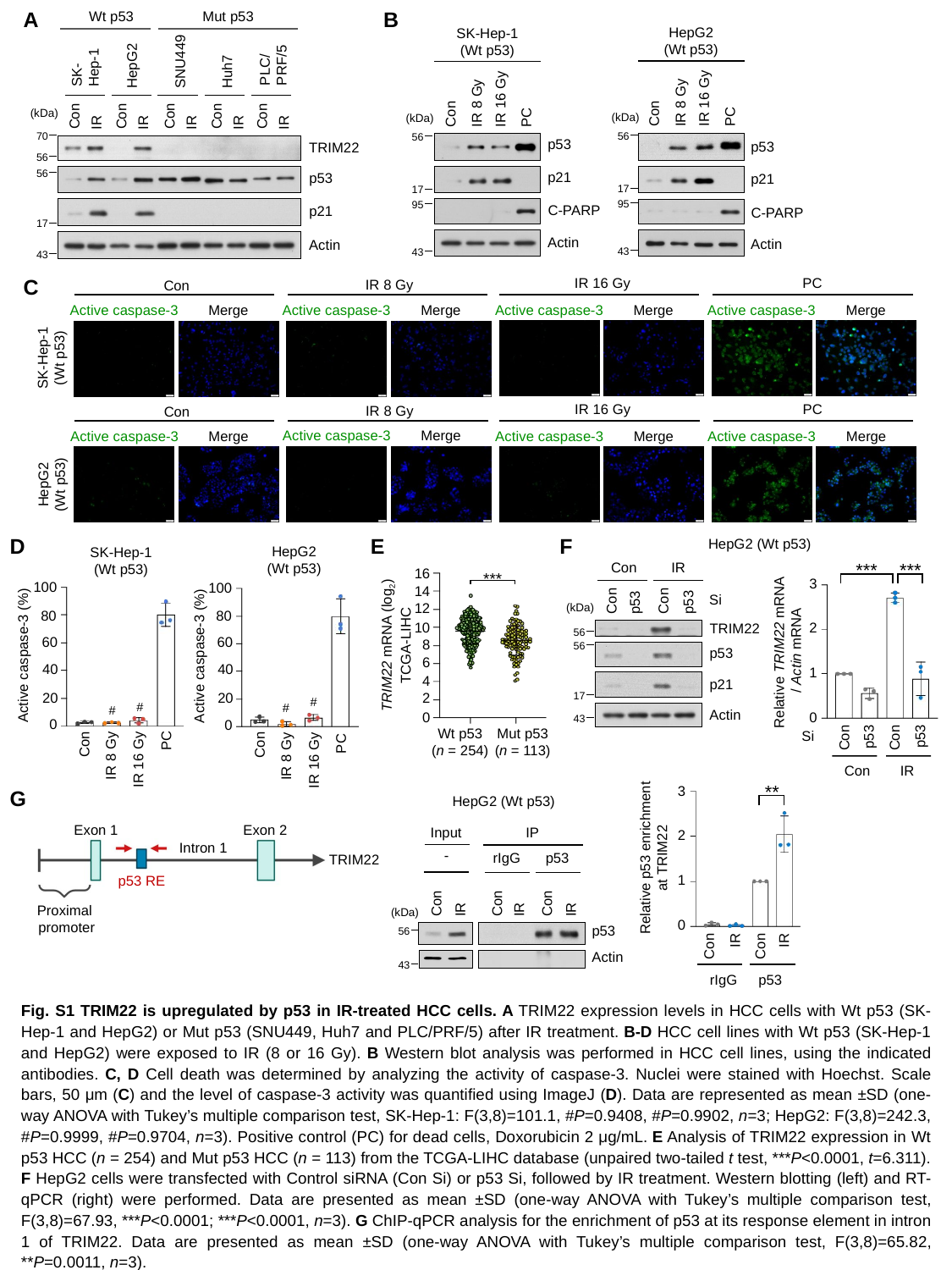

A
B
Wt p53
Mut p53
PLC/
PRF/5
SK-
Hep-1
SNU449
HepG2
Huh7
Con
Con
Con
Con
Con
IR
IR
IR
IR
IR
TRIM22
p53
p21
Actin
HepG2
(Wt p53)
IR 16 Gy
IR 8 Gy
Con
PC
56
p53
p21
17
95
C-PARP
Actin
43
(kDa)
SK-Hep-1
(Wt p53)
IR 16 Gy
IR 8 Gy
Con
PC
(kDa)
56
p53
p21
17
95
C-PARP
Actin
43
(kDa)
70
56
56
17
43
C
IR 16 Gy
PC
IR 8 Gy
Con
Active caspase-3
Merge
Active caspase-3
Merge
Active caspase-3
Merge
Active caspase-3
Merge
IR 16 Gy
PC
IR 8 Gy
Con
Active caspase-3
Merge
Active caspase-3
Merge
Active caspase-3
Merge
Active caspase-3
Merge
SK-Hep-1
(Wt p53)
HepG2
(Wt p53)
D
E
F
HepG2 (Wt p53)
***
***
Con
IR
Si
Con
Con
p53
p53
TRIM22
p53
p21
Actin
3
(kDa)
2
56
Relative TRIM22 mRNA
 / Actin mRNA
56
1
17
0
43
Si
p53
p53
Con
Con
Con
IR
HepG2
(Wt p53)
SK-Hep-1
(Wt p53)
16
***
14
12
10
TRIM22 mRNA (log2)
TCGA-LIHC
8
6
4
2
0
Wt p53
(n = 254)
Mut p53
(n = 113)
100
80
60
Active caspase-3 (%)
40
20
#
#
0
PC
Con
IR 8 Gy
IR 16 Gy
100
80
60
Active caspase-3 (%)
40
20
#
#
0
PC
Con
IR 8 Gy
IR 16 Gy
**
3
2
Relative p53 enrichment
at TRIM22
1
0
IR
IR
Con
Con
rIgG
p53
G
HepG2 (Wt p53)
Exon 1
Exon 2
Intron 1
TRIM22
p53 RE
Proximal
promoter
Input
IP
-
rIgG
p53
Con
Con
Con
IR
IR
IR
p53
Actin
(kDa)
56
43
Fig. S1 TRIM22 is upregulated by p53 in IR-treated HCC cells. A TRIM22 expression levels in HCC cells with Wt p53 (SK-Hep-1 and HepG2) or Mut p53 (SNU449, Huh7 and PLC/PRF/5) after IR treatment. B-D HCC cell lines with Wt p53 (SK-Hep-1 and HepG2) were exposed to IR (8 or 16 Gy). B Western blot analysis was performed in HCC cell lines, using the indicated antibodies. C, D Cell death was determined by analyzing the activity of caspase-3. Nuclei were stained with Hoechst. Scale bars, 50 μm (C) and the level of caspase-3 activity was quantified using ImageJ (D). Data are represented as mean ±SD (one-way ANOVA with Tukey’s multiple comparison test, SK-Hep-1: F(3,8)=101.1, #P=0.9408, #P=0.9902, n=3; HepG2: F(3,8)=242.3, #P=0.9999, #P=0.9704, n=3). Positive control (PC) for dead cells, Doxorubicin 2 μg/mL. E Analysis of TRIM22 expression in Wt p53 HCC (n = 254) and Mut p53 HCC (n = 113) from the TCGA-LIHC database (unpaired two-tailed t test, ***P<0.0001, t=6.311). F HepG2 cells were transfected with Control siRNA (Con Si) or p53 Si, followed by IR treatment. Western blotting (left) and RT-qPCR (right) were performed. Data are presented as mean ±SD (one-way ANOVA with Tukey’s multiple comparison test, F(3,8)=67.93, ***P<0.0001; ***P<0.0001, n=3). G ChIP-qPCR analysis for the enrichment of p53 at its response element in intron 1 of TRIM22. Data are presented as mean ±SD (one-way ANOVA with Tukey’s multiple comparison test, F(3,8)=65.82, **P=0.0011, n=3).

## Slide 2
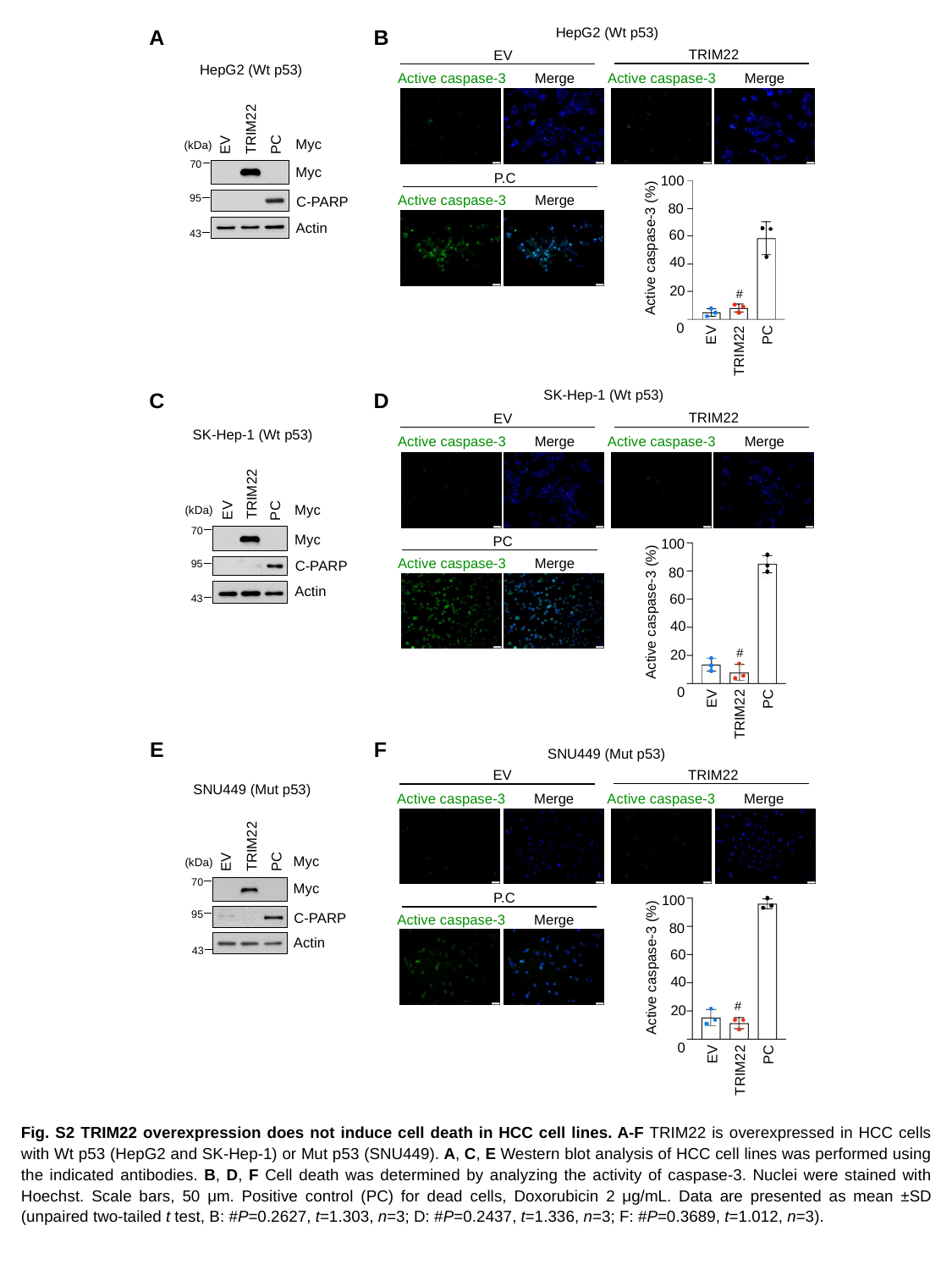

HepG2 (Wt p53)
TRIM22
EV
Active caspase-3
Merge
Active caspase-3
Merge
P.C
Active caspase-3
Merge
100
80
60
Active caspase-3 (%)
40
20
#
0
EV
PC
TRIM22
A
B
HepG2 (Wt p53)
TRIM22
Myc
PC
EV
(kDa)
70
Myc
95
C-PARP
Actin
43
SK-Hep-1 (Wt p53)
TRIM22
EV
Active caspase-3
Merge
Active caspase-3
Merge
PC
Active caspase-3
Merge
100
80
60
Active caspase-3 (%)
40
#
20
0
EV
PC
TRIM22
C
D
SK-Hep-1 (Wt p53)
TRIM22
Myc
EV
PC
(kDa)
70
Myc
C-PARP
95
Actin
43
E
F
SNU449 (Mut p53)
TRIM22
EV
SNU449 (Mut p53)
Active caspase-3
Merge
Active caspase-3
Merge
TRIM22
Myc
PC
EV
(kDa)
70
Myc
P.C
100
95
C-PARP
Active caspase-3
Merge
80
Actin
43
60
Active caspase-3 (%)
40
#
20
0
EV
PC
TRIM22
Fig. S2 TRIM22 overexpression does not induce cell death in HCC cell lines. A-F TRIM22 is overexpressed in HCC cells with Wt p53 (HepG2 and SK-Hep-1) or Mut p53 (SNU449). A, C, E Western blot analysis of HCC cell lines was performed using the indicated antibodies. B, D, F Cell death was determined by analyzing the activity of caspase-3. Nuclei were stained with Hoechst. Scale bars, 50 μm. Positive control (PC) for dead cells, Doxorubicin 2 μg/mL. Data are presented as mean ±SD (unpaired two-tailed t test, B: #P=0.2627, t=1.303, n=3; D: #P=0.2437, t=1.336, n=3; F: #P=0.3689, t=1.012, n=3).

## Slide 3
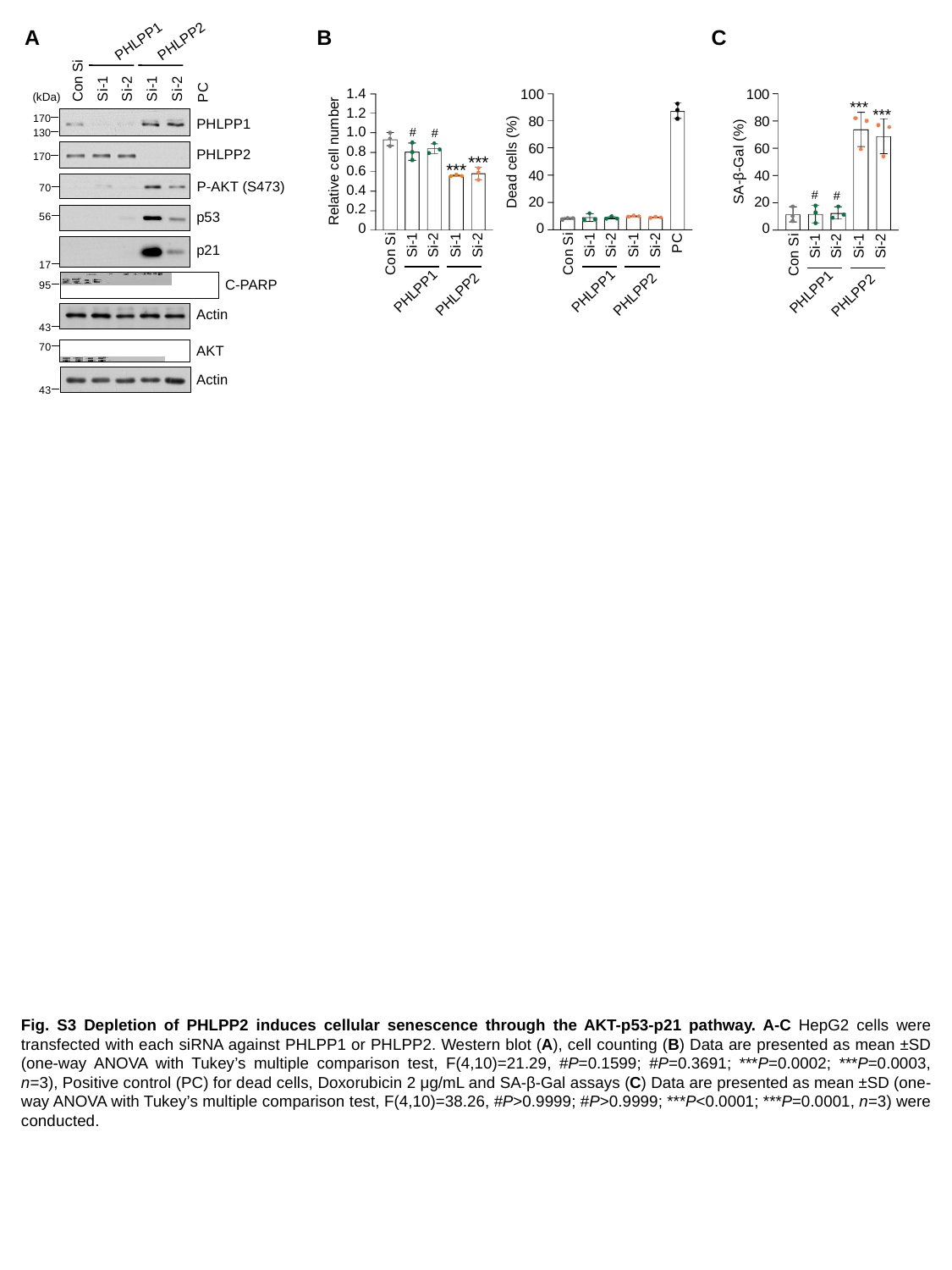

A
B
C
PHLPP1
PHLPP2
Con Si
Si-1
Si-2
Si-1
Si-2
PC
1.4
100
100
(kDa)
***
1.2
***
170
80
80
PHLPP1
1.0
#
#
130
60
60
0.8
PHLPP2
170
***
Relative cell number
SA-β-Gal (%)
Dead cells (%)
***
0.6
40
40
P-AKT (S473)
0.4
70
#
#
20
20
0.2
p53
56
0
0
0
PC
Si-1
Si-2
Si-1
Si-2
Si-1
Si-2
Si-1
Si-2
Si-1
Si-2
Si-1
Si-2
p21
Con Si
Con Si
Con Si
17
C-PARP
95
PHLPP1
PHLPP1
PHLPP1
PHLPP2
PHLPP2
PHLPP2
Actin
43
70
AKT
Actin
43
Fig. S3 Depletion of PHLPP2 induces cellular senescence through the AKT-p53-p21 pathway. A-C HepG2 cells were transfected with each siRNA against PHLPP1 or PHLPP2. Western blot (A), cell counting (B) Data are presented as mean ±SD (one-way ANOVA with Tukey’s multiple comparison test, F(4,10)=21.29, #P=0.1599; #P=0.3691; ***P=0.0002; ***P=0.0003, n=3), Positive control (PC) for dead cells, Doxorubicin 2 μg/mL and SA-β-Gal assays (C) Data are presented as mean ±SD (one-way ANOVA with Tukey’s multiple comparison test, F(4,10)=38.26, #P>0.9999; #P>0.9999; ***P<0.0001; ***P=0.0001, n=3) were conducted.

## Slide 4
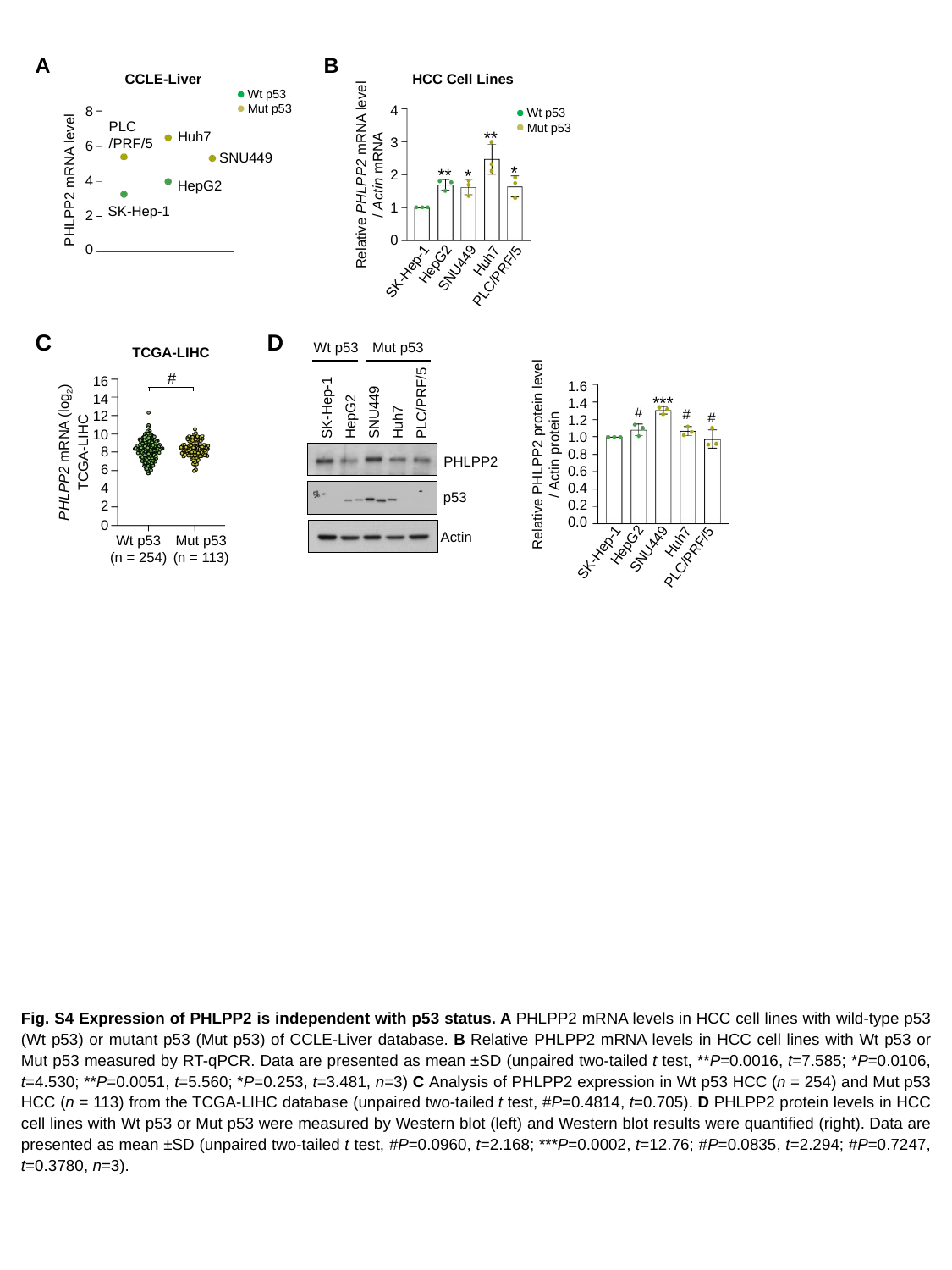

A
B
CCLE-Liver
HCC Cell Lines
Wt p53
Mut p53
4
8
Wt p53
Mut p53
PLC
/PRF/5
**
Huh7
3
6
SNU449
Relative PHLPP2 mRNA level
/ Actin mRNA
*
**
*
2
PHLPP2 mRNA level
4
HepG2
1
SK-Hep-1
2
0
0
Huh7
HepG2
SNU449
SK-Hep-1
PLC/PRF/5
C
D
Wt p53
Mut p53
PLC/PRF/5
SK-Hep-1
SNU449
HepG2
Huh7
PHLPP2
p53
Actin
TCGA-LIHC
1.6
***
1.4
#
#
#
1.2
1.0
Relative PHLPP2 protein level
/ Actin protein
0.8
0.6
0.4
0.2
0.0
Huh7
HepG2
SNU449
SK-Hep-1
PLC/PRF/5
#
16
14
12
10
PHLPP2 mRNA (log2)
TCGA-LIHC
8
6
4
2
0
Wt p53
(n = 254)
Mut p53
(n = 113)
Fig. S4 Expression of PHLPP2 is independent with p53 status. A PHLPP2 mRNA levels in HCC cell lines with wild-type p53 (Wt p53) or mutant p53 (Mut p53) of CCLE-Liver database. B Relative PHLPP2 mRNA levels in HCC cell lines with Wt p53 or Mut p53 measured by RT-qPCR. Data are presented as mean ±SD (unpaired two-tailed t test, **P=0.0016, t=7.585; *P=0.0106, t=4.530; **P=0.0051, t=5.560; *P=0.253, t=3.481, n=3) C Analysis of PHLPP2 expression in Wt p53 HCC (n = 254) and Mut p53 HCC (n = 113) from the TCGA-LIHC database (unpaired two-tailed t test, #P=0.4814, t=0.705). D PHLPP2 protein levels in HCC cell lines with Wt p53 or Mut p53 were measured by Western blot (left) and Western blot results were quantified (right). Data are presented as mean ±SD (unpaired two-tailed t test, #P=0.0960, t=2.168; ***P=0.0002, t=12.76; #P=0.0835, t=2.294; #P=0.7247, t=0.3780, n=3).

## Slide 5
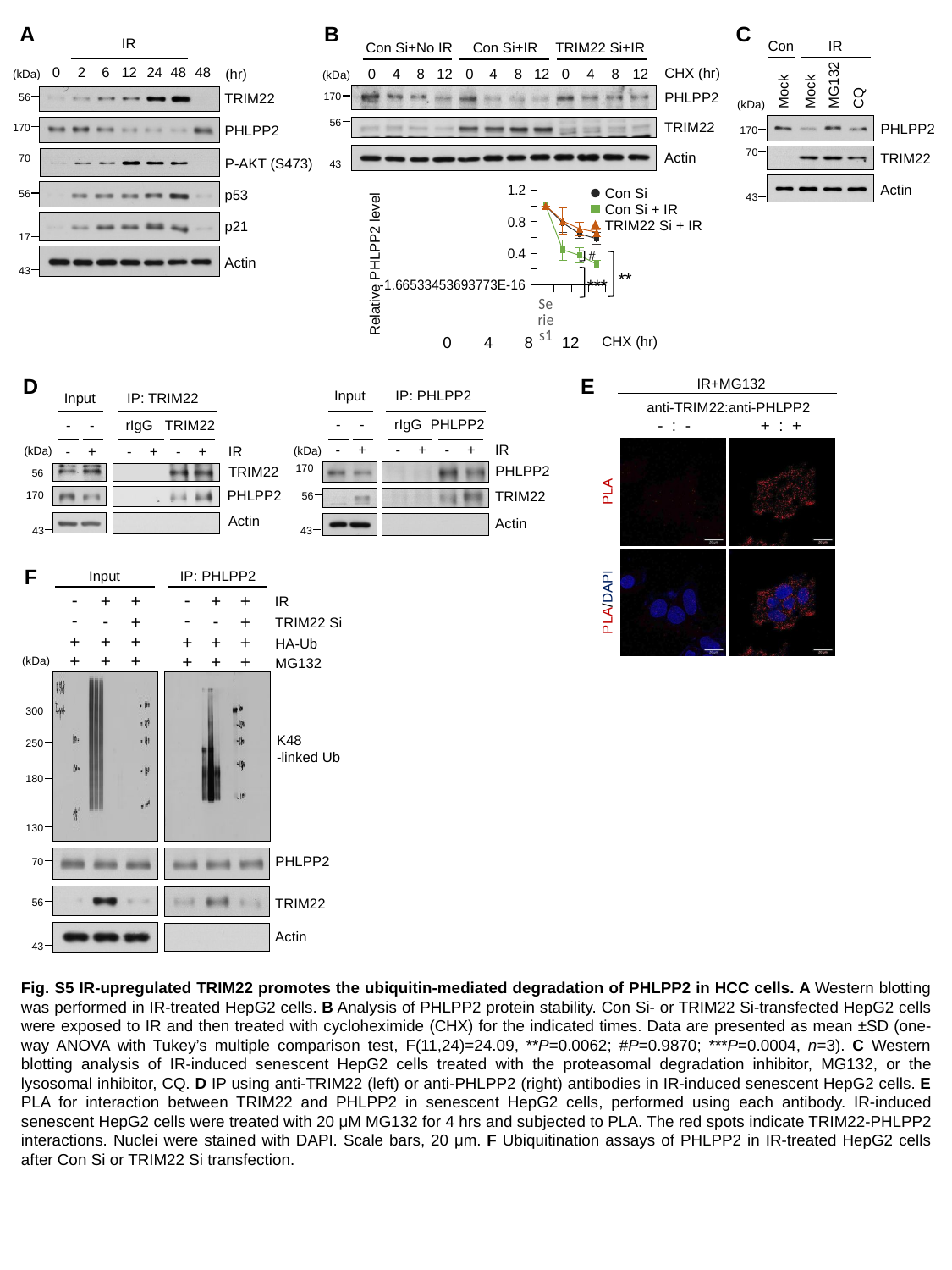

A
B
C
IR
0
2
6
12
24
48
48
(hr)
TRIM22
PHLPP2
P-AKT (S473)
p53
p21
Actin
Con
IR
MG132
Mock
Mock
CQ
PHLPP2
TRIM22
Actin
Con Si+No IR
Con Si+IR
TRIM22 Si+IR
CHX (hr)
0
4
8
12
0
4
8
12
0
4
8
12
PHLPP2
TRIM22
Actin
(kDa)
(kDa)
170
56
(kDa)
56
170
170
70
70
43
Con Si
Con Si + IR
TRIM22 Si + IR
### Chart
| Category | | | |
|---|---|---|---|
| | 1.0 | 1.0 | 1.0 |
| | 0.7845498971581076 | 0.4437567595758069 | 0.8087316027227031 |
| | 0.6464673252641002 | 0.37370369718639235 | 0.7098594500815668 |
| | 0.5840246524340502 | 0.2630523102128148 | 0.6668147485638526 |#
Relative PHLPP2 level
**
***
CHX (hr)
0
4
8
12
56
43
17
43
D
E
IR+MG132
Input
IP: PHLPP2
-
-
rIgG
PHLPP2
IR
-
+
-
+
-
+
PHLPP2
TRIM22
Actin
IP: TRIM22
Input
anti-TRIM22:anti-PHLPP2
- : -
+ : +
-
-
rIgG
TRIM22
-
+
-
+
-
+
IR
(kDa)
(kDa)
170
TRIM22
56
PLA
PHLPP2
170
56
Actin
43
43
F
Input
IP: PHLPP2
-
-
+
+
+
+
IR
-
-
-
-
+
+
TRIM22 Si
+
+
+
+
+
+
HA-Ub
+
+
+
+
+
+
(kDa)
MG132
300
K48
-linked Ub
250
180
130
PHLPP2
70
TRIM22
56
Actin
43
PLA/DAPI
Fig. S5 IR-upregulated TRIM22 promotes the ubiquitin-mediated degradation of PHLPP2 in HCC cells. A Western blotting was performed in IR-treated HepG2 cells. B Analysis of PHLPP2 protein stability. Con Si- or TRIM22 Si-transfected HepG2 cells were exposed to IR and then treated with cycloheximide (CHX) for the indicated times. Data are presented as mean ±SD (one-way ANOVA with Tukey’s multiple comparison test, F(11,24)=24.09, **P=0.0062; #P=0.9870; ***P=0.0004, n=3). C Western blotting analysis of IR-induced senescent HepG2 cells treated with the proteasomal degradation inhibitor, MG132, or the lysosomal inhibitor, CQ. D IP using anti-TRIM22 (left) or anti-PHLPP2 (right) antibodies in IR-induced senescent HepG2 cells. E PLA for interaction between TRIM22 and PHLPP2 in senescent HepG2 cells, performed using each antibody. IR-induced senescent HepG2 cells were treated with 20 μM MG132 for 4 hrs and subjected to PLA. The red spots indicate TRIM22-PHLPP2 interactions. Nuclei were stained with DAPI. Scale bars, 20 μm. F Ubiquitination assays of PHLPP2 in IR-treated HepG2 cells after Con Si or TRIM22 Si transfection.

## Slide 6
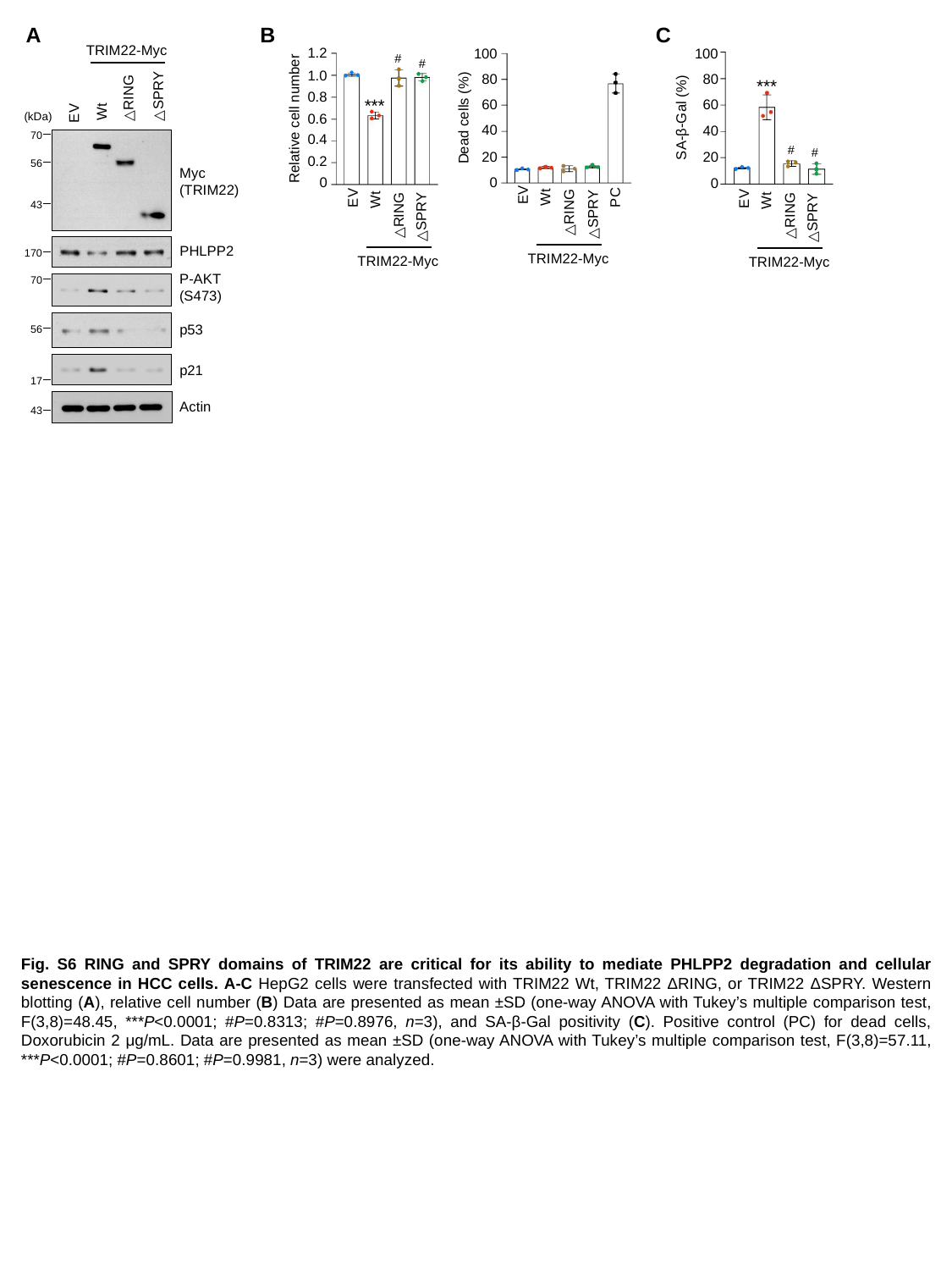

A
B
C
100
80
60
Dead cells (%)
40
20
0
EV
Wt
PC
△RING
△SPRY
TRIM22-Myc
1.2
#
#
1.0
0.8
***
0.6
Relative cell number
0.4
0.2
0
EV
Wt
△RING
△SPRY
TRIM22-Myc
TRIM22-Myc
100
80
***
60
SA-β-Gal (%)
40
#
#
20
0
EV
Wt
△RING
△SPRY
TRIM22-Myc
△SPRY
△RING
Wt
EV
(kDa)
70
56
Myc
(TRIM22)
43
PHLPP2
170
P-AKT
(S473)
70
p53
56
p21
17
Actin
43
Fig. S6 RING and SPRY domains of TRIM22 are critical for its ability to mediate PHLPP2 degradation and cellular senescence in HCC cells. A-C HepG2 cells were transfected with TRIM22 Wt, TRIM22 ΔRING, or TRIM22 ΔSPRY. Western blotting (A), relative cell number (B) Data are presented as mean ±SD (one-way ANOVA with Tukey’s multiple comparison test, F(3,8)=48.45, ***P<0.0001; #P=0.8313; #P=0.8976, n=3), and SA-β-Gal positivity (C). Positive control (PC) for dead cells, Doxorubicin 2 μg/mL. Data are presented as mean ±SD (one-way ANOVA with Tukey’s multiple comparison test, F(3,8)=57.11, ***P<0.0001; #P=0.8601; #P=0.9981, n=3) were analyzed.

## Slide 7
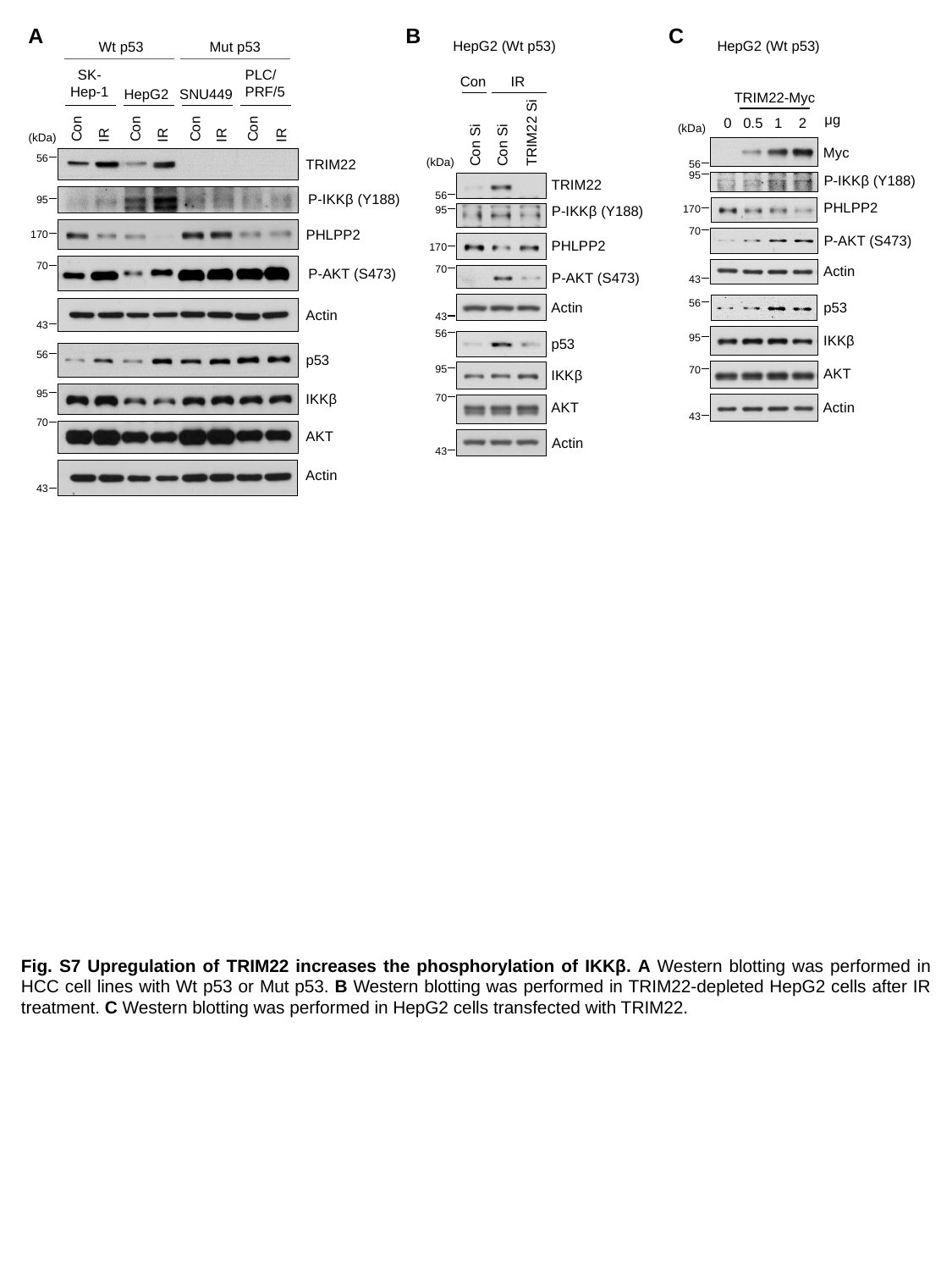

A
B
C
HepG2 (Wt p53)
HepG2 (Wt p53)
Wt p53
Mut p53
SK-
Hep-1
PLC/
PRF/5
Con
IR
HepG2
SNU449
TRIM22-Myc
μg
0
0.5
1
2
Con
Con
Con
Con
(kDa)
TRIM22 Si
IR
IR
IR
IR
(kDa)
Con Si
Con Si
Myc
56
(kDa)
TRIM22
56
95
P-IKKβ (Y188)
TRIM22
56
P-IKKβ (Y188)
95
PHLPP2
P-IKKβ (Y188)
170
95
70
PHLPP2
170
P-AKT (S473)
PHLPP2
170
70
70
Actin
P-AKT (S473)
P-AKT (S473)
43
56
Actin
p53
Actin
43
43
56
95
IKKβ
p53
56
p53
95
70
AKT
IKKβ
95
IKKβ
70
Actin
AKT
43
70
AKT
Actin
43
Actin
43
Fig. S7 Upregulation of TRIM22 increases the phosphorylation of IKKβ. A Western blotting was performed in HCC cell lines with Wt p53 or Mut p53. B Western blotting was performed in TRIM22-depleted HepG2 cells after IR treatment. C Western blotting was performed in HepG2 cells transfected with TRIM22.
